# An improved Differential evolution with Sailfish optimizer (DESFO) for handling feature selection problem

**DOI:** 10.1038/s41598-024-63328-w

**Published:** 2024-06-12

**Authors:** Safaa. M. Azzam, O. E. Emam, Ahmed Sabry Abolaban

**Affiliations:** Department of Information Systems, Faculty of Computers and Artificial Intelligence, Helwan University, P.O. Box 11795, Helwan, Egypt

**Keywords:** Feature selection, Optimization, Meta-heuristics, Local search, Classification, Machine learning, Swam intelligence, Differential evolution, Sailfish, Exploration, Exploitation, Applied mathematics, Computational science, Computer science, Information technology, Statistics, Information theory and computation

## Abstract

As a preprocessing for machine learning and data mining, Feature Selection plays an important role. Feature selection aims to streamline high-dimensional data by eliminating irrelevant and redundant features, which reduces the potential curse of dimensionality of a given large dataset. When working with datasets containing many features, algorithms that aim to identify the most valuable features to improve dataset accuracy may encounter difficulties because of local optima. Many studies have been conducted to solve this problem. One of the solutions is to use meta-heuristic techniques. This paper presents a combination of the Differential evolution and the sailfish optimizer algorithms (DESFO) to tackle the feature selection problem. To assess the effectiveness of the proposed algorithm, a comparison between Differential Evolution, sailfish optimizer, and nine other modern algorithms, including different optimization algorithms, is presented. The evaluation used Random forest and key nearest neighbors as quality measures. The experimental results show that the proposed algorithm is a superior algorithm compared to others. It significantly impacts high classification accuracy, achieving 85.7% with the Random Forest classifier and 100% with the Key Nearest Neighbors classifier across 14 multi-scale benchmarks. According to fitness values, it gained 71% with the Random forest and 85.7% with the Key Nearest Neighbors classifiers.

## Introduction

Recently, the swift progress in high-throughput technologies has resulted in a significant growth in data, both in its complexity and the volume of samples. The challenge of managing this extensive and intricate data efficiently is becoming more pronounced. The conventional manual approaches to dealing with these data sets are now considered unfeasible. Consequently, data mining (DM) and machine learning (ML) methods have risen to the forefront, offering automated knowledge extraction and pattern identification solutions within this vast data.

A notable obstacle encountered in this procedure is the prevalent noise within the gathered data. This noise can result from multiple factors, including imperfections in the data collection technologies and the data sources’ intrinsic characteristics. For example, in medical imaging, any malfunction in the imaging devices can lead to noise in the data, which can interfere with further analysis. Furthermore, the rise of social media has shifted online users from merely consuming content to producing and consuming it. The quality of data from social media platforms can vary dramatically, from extremely valuable to spam or offensive content. Additionally, social media data often features informal language characterized by grammatical mistakes, typos, and incorrect punctuation. This diversity and lack of formality increase the difficulty of deriving meaningful knowledge and patterns from such broad and noisy datasets. In the process of classification for machine learning and Data mining, the primary aim is to identify the category of each instance in a given dataset using a two-phase approach—training and testing. For this goal, the classifier model is created during the training phase to classify each instance in the training set, which consists of available records. During the later stages of testing, the classifier’s precision is evaluated using a group of testing sets. These sets were not employed during the training phase, but this research concerns their respective classes. Dealing with high dimensionality can pose a significant obstacle and may hinder the effectiveness of the classification process. Datasets containing many features may be utilized in specific practical applications and fields, such as the medical field, bioinformatics, text mining, and image classification. However, some of these features may need to be more relevant, redundant, or contain noise. Such characteristics in the dataset could result in over-fitting data or create ambiguity in the learning mechanism^1,2^.

Feature Selection (FS) is commonly employed as a prepossessing step to improve the accuracy of a classification model. The core objective of FS is to identify the most relevant features that positively impact model performance while discarding irrelevant or harmful features at a minimal cost^3^. Various algorithms have been created to identify the most effective set of features that can improve the accuracy of a classification model for a given dataset. When dealing with datasets containing many features, traditional algorithms encounter challenges in identifying the significant features.

There are three FS (Feature Selection) algorithm types: filter, wrapper, and embedding. Regarding filtering algorithms, the FS process and classifier model are treated as distinct phases. During the initial phase, specific metrics extract features from the dataset that significantly impact the classification process while ignoring the others. In the feature selection process, only the chosen attributes are used in the classification model for its phase. However, wrapper algorithms modify the selected feature subsets dynamically, depending on the accuracy of the classifier. In Feature Selection (FS), the wrapper approach is commonly used. This approach involves generating subsets of features using specific search methods and determining their relevance by running a classification algorithm. Embedded algorithms are then combined with a classifier to decide which features should be kept or removed from the dataset^4–6^.

As per reference^7^, FS is widely believed to present a combinatorial optimization problem that is most likely NP-complete. Each feature in a dataset has twice as many potential solutions, making it challenging and time-consuming to determine the most efficient subset of features. Additionally, in references^8,9^, the feature selection (FS) problem is a problem in the field of optimization that is considered to be NP-hard. This means that the more complex the problem, the longer it takes to compute the solution, with computational time increasing exponentially. Hence, researchers have shown a keen interest in meta-heuristic (MH) algorithms^10^; four main categories of algorithms excel in solving various optimization problems. These categories include Human-based algorithms, Swarm intelligence algorithms (SI), Physics-based algorithms (PA), and Evolutionary Algorithms (EA).

Swarms and animal behavioral patterns are the basis for SI algorithms^11^. A commonly employed algorithm in optimization problems is Particle Swarm Optimization (PSO). The algorithm is designed based on the collective behaviors of swarm objects. In this approach, every individual object represents a potential solution^12^. The concept behind Artificial Fish Swarm (AFS) involves replicating the actions of fish, such as hunting, gathering in groups, and tracking, to perform a localized search of individuals to attain a global optimal solution. This technique is discussed in reference^13^. Bacterial Foraging Optimization (BFO) is a recently developed algorithm that draws inspiration from the foraging behavior of Escherichia coli in humans. It involves competition and cooperation among bacterial populations and is employed as a global random search algorithm^14^. Ant Colony Optimization (ACO) is a well-known swarm intelligence algorithm that imitates the foraging behavior of different ant species. In natural settings, ants use chemical pheromones to identify the most optimal path for the colony members to follow^15^. A swarm intelligence optimizer known as pigeon-inspired optimization solves air-robot path planning problems. The technique involves using a map and compass operator model based on a magnetic field and the sun and a landmark operator model that utilizes landmarks^16^. The bat algorithm is a metaheuristic algorithm based on the behavior of animal groups or herds. It uses the echolocation behavior of bats to generate solutions for domains with single- or multi-objectives that exist within a continuous solution space. This information is based on reference^17^. The grey wolf optimizer is an algorithm that imitates the leadership hierarchy and hunting mechanisms of grey wolves in nature and is categorized as a swarm intelligence algorithm^18^.

To effectively search a given space, any search algorithm must balance exploring new areas within that space with exploiting already known areas. This means it must balance venturing into uncharted territory and focusing on areas near previously explored locations. By achieving an optimal balance between exploration and exploitation, a search algorithm is more likely to succeed in its search efforts^19^.

There have been multiple attempts to understand the mechanism that regulates the equilibrium between exploration and exploitation in search algorithms. However, due to the need for more consistent knowledge, several interesting metrics have been proposed to quantify the level of exploration and exploitation in metaheuristic schemes. These metrics monitor the current diversity of the population and have been suggested in various indexes. Despite several indexes and ongoing proposals, there is yet to be a definitive or objective way to measure metaheuristic algorithms' exploration/exploitation rate^20^. Achieving success with metaheuristic algorithms requires a careful balance between exploration and exploitation throughout the evolutionary process. To achieve this balance more effectively, it is important to optimize the level of exploration and exploitation^21^.

Many SI algorithms that show high performance in various optimization problems have been developed in the literature. Some of these algorithms include the sailfish optimizer (SFO)^22^, Chaotic Coyote Algorithm^23^, Modified Social-Spider Optimization Algorithm^24^, Cheetah Optimization Algorithm^25^, Migrating Birds Optimization^26^, Owl Optimization Algorithm^27^, Bacterial Foraging Optimization Algorithm^28^, Salp Swarm Algorithm (SSA)^29^.

Many metaheuristic algorithms are based on evolutionary behaviors that emulate biological processes such as mutation, crossover, and selection, and they are named EA algorithms. Some of these algorithms include Differential Evolution (DE)^30^, Genetic Algorithm (GA)^31^, Invasive Tumor Growth Optimizer (ITGO)^32^ and Biogeography-Based Optimizer (BBO)^33^.These algorithms have shown great efficiency in various optimization applications.

Optimization algorithms that are based on physical laws are called PhA algorithms and include Big Bang-Big Crunch BBBC^34^, Multi-verse Optimizer (MVO)^35^, and Gravitational Search Algorithm (GSA)^36^.

### Contribution

The proposed framework in this paper puts forward a hybrid algorithm that combines the DE algorithm with the SFO algorithm to handle the FS strategy. It offers novel contributions that can be summarized as follows:

1. A new algorithm called the DESFO algorithm has been created by integrating and reproducing DE and SFO.
2. The transfer function (TF) is the V-shaped function to convert position values into binary format.
3. The periodic mode boundary handling (PMBH) approach and a novel local search (LS) strategy are used to improve the exploration and exploitation process.
4. In supervised classification, the DESFO algorithm is used for wrapper feature selection.
5. The DESFO’s performance is evaluated through metrics such as average fitness rate, average accuracy rate, and average number of selected features.
6. To assess the effectiveness of the suggested DESFO algorithm with the RF and K-NN machine classification algorithms, a Wilcoxon's non-parametric rank-sum test (with a significance level of 5%) is used to compare it with similar algorithms.

### Structure

The paper follows the structure outlined below:

1. Section “Related works” provides the recent stats of art and related works.
2. Section “Preliminary work” provides Preliminary works and explanations about the original DE and SFO algorithms.
3. Section “Methodology of the proposed DESFO” introduces the methodology of the proposed algorithm DESFO, along with the related steps.
4. Section “Experimental results and analysis” presents the experimental results of the DESFO algorithm and compares it with other MH algorithms.
5. Section “Conclusion and future works” concludes the paper.

## Related works

Numerous research studies have been conducted in feature selection utilizing metaheuristic algorithms. Some of these efforts are outlined below.

Rodrigues et al.^37^ introduced a binary cuckoo search algorithm called BCS, which uses a function to convert continuous variables to their binary form to obtain the optimal feature subset. The Optimum Path Forest classifier was used to apply BCS on two datasets related to theft detection in a power system. The results indicated that BCS was the most efficient and appropriate method for solving feature selection issues in industrial datasets while also being the fastest.

In their study, Emary et al.^38^ introduced the initial binary edition of the firefly algorithm (FFA) for addressing feature selection issues by utilizing a threshold value. The algorithm exhibited a high level of exploration quality, enabling it to swiftly identify a solution to the problem.

To tackle feature selection problems, Nakamura et al.^39^ developed a binary version of BA called BBA. They used a sigmoid function to confine the position of bats to binary variables. They employed the optimum path forest classifier and applied BBA to five datasets to evaluate the accuracy.

Zawbaa et al.^40^ proposed a binary version of the ALO algorithm to address the feature selection problem by applying a threshold value to continuous variables. In their study, Emary et al.^41^ employed the sigmoidal transfer function to obtain binary vectors, also known as bGWO. They evaluated the classification accuracy of these vectors using a K-NN classifier across eighteen distinct UCI datasets. The researchers also utilized small, random, and large initialization methods during the initialization phase to facilitate thorough exploration.

Hussien et al.^42,43^ utilized S and V-shaped transfer functions in conventional WOA to solve binary optimization problems. They also applied this method to solve feature selection problems with eleven UCI datasets. To ensure the relevance of the selected features for classification, they used the K-NN classifier.

In their study, Gad et al.^44^ introduced a new version of the sparrow search algorithm, which has been developed. This version uses a combination of random agent repositioning and the LS method to handle feature selection effectively in supervised classification tasks. This approach is particularly useful for choosing the best or nearly optimal subset of attributes from a given dataset while maintaining maximum accuracy rates.

Ghosh et al.^45^ have presented a new variant of the latest and most powerful optimizer, the Sailfish Optimizer (SFO), called the Binary Sailfish (BSF) optimizer for solving FS problems. They utilized the sigmoid transfer function to convert the continuous search space of SFO into a binary one. They also incorporated adaptive β-hill climbing (AβHC), a recently proposed meta-heuristic algorithm, with the BSF optimizer to enhance its exploitation ability.

Emrah et al.^46^ have proposed a new filter criterion that mutual information, ReliefF, and Fisher Score inspire. Rather than relying on mutual redundancy, this criterion aims to select the most highly ranked features determined by Relief and Fisher Score while ensuring mutual relevance between the features and class labels. Based on this new criterion, the team has developed two novel differential evolution (DE) based filter approaches.

Bacanin et al.^47^, presented a diversity-oriented social network search to tackle the feature selection problem in detecting phishing websites. The authors aimed to enhance the detection of phishing websites by refining an extreme learning model that leverages the most pertinent subset of features from the phishing websites dataset. A new algorithm was developed and integrated into a two-level cooperative framework to accomplish this. The efficacy of the proposed algorithm was then evaluated and compared against six other state-of-the-art metaheuristics algorithms.

Alrefai et al.^48^ Proposed an effective method for cancer classification using ensemble learning. The study employed particle swarm optimization and an ensemble learning method for feature selection and cancer classification. The study's findings indicate that the proposed method is effective for cancer classification based on microarray datasets. Furthermore, the accuracy of the proposed method proves its superiority over other methods.

Gomez et al ^49^ proposed a new technique called Two-Step Swarm Intelligence. The method involves breaking down the heuristic search carried out by agents into two stages. In the first phase, agents generate partial solutions, used as starting states in the second phase. Our study aimed to assess the effectiveness of this approach in resolving the Feature Selection Problem using Ant Colony Optimization and Particle Swarm Optimization. The feature selection is based on the reduction concept in the Rough Set Theory. The results demonstrate that the Two-Step Swarm Intelligence method improves the performance of ACO and PSO metaheuristics regarding computation time and the quality of reduction produced.

Bezdan et al.^50^ proposed an algorithm based on a binary hybrid metaheuristic approach to select the optimal feature subset. Specifically, they combined the brainstorm optimization algorithm with the firefly algorithm to create a wrapper method for feature selection problems on classification data sets. The performance of the proposed algorithm was evaluated on 21 data sets and compared against 11 other metaheuristic algorithms. Additionally, the algorithm was applied to the coronavirus data set.

Gao et al.^51^ Introduced a Clustering Probabilistic Particle Swarm Optimization (CPPSO) to improve the traditional particle swarm optimization approach. CPPSO incorporates probabilities to represent velocity and an elitism mechanism. Additionally, CPPSO uses the K-means algorithm to cluster the population based on the Hamming distance into two sub-populations, which enhances its performance. The effectiveness of CPPSO is evaluated by comparing it against seven existing algorithms using twenty diverse datasets.

Latha et al.^52^ Addressed the feature selection problem by implementing grey wolf optimization (GWO) with decomposed random differential grouping (DrnDG-GWO) as a supervised learning technique. The study found that combining supervised machine learning with swarm intelligence techniques yielded the best feature optimization results.

### Motivations

Storn et al.^30^ proposed the differential evolution (DE) algorithm in 1997, a powerful and straightforward stochastic search method operating on populations. DE is an effective global optimizer for continuous search problems and has been successfully applied in various domains, such as pattern recognition^53^, communication^54^, and mechanical engineering^55,56^.

The Sailfish Optimizer (SFO) is a highly effective optimization algorithm developed and presented in 2019 by a team of researchers known as Shadravan et al.^22^. This algorithm is based on the concept of population, and it mimics the hunting behavior of a group of sailfish as they hunt for a school of sardines. The strategy employed by the sailfish group involves alternating between attacking a group of sardines and retreating to capture their prey. The SFO algorithm has become popular in the optimization community due to its robustness and effectiveness. In this paper, an algorithm called DESFO that integrates both DE and SFO has been proposed. Due to their power and superiority, the proposed algorithm can attain satisfactory search accuracy, swift convergence speed, and improved stability.

Moreover, it can prevent getting stuck in local optima, which is an issue that still needs to be systematically addressed for the FS problem. On the other hand, compared to the state-of-the-art meta-heuristic techniques, including the original DE and SFO, the DESFO approach yields superior results by producing optimal or near-optimal outcomes for numerous problems. The proposed feature selection algorithm method was tested on 14 benchmarks using multi-scale attributes and records from the UCI machine learning repository. This implementation was carried out 30 times to validate its efficacy^57^. The average classification accuracy is calculated using two standard machine learning classification algorithms: Random Forest (RF) and k-nearest Neighbor (k-NN).

## Preliminary work

As mentioned in the previous section, meta-heuristics have several benefits, but can existing methods adequately solve the FS problem? The No Free Lunch theorem (NFL)^58^ answers this question. This theorem suggests that no single algorithm can perfectly solve all optimization problems. In the case of FS on a dataset, an algorithm may perform exceptionally well for one dataset but inadequately for another. Therefore, there is still a need for an advanced metaheuristic approach that can efficiently solve almost all possible FS dataset types, which is currently an open research question. From this point in this section of the paper, the basic DE algorithm and SFO algorithm will be explained. The two algorithms will be integrated under the DESFO algorithm to optimize the feature selection problem and enhance classification accuracy.

### Differential evolution algorithm (DE)

In 1997, Storn et al.^30^ introduced a Differential Evolution (DE) algorithm, considered one of the most reliable versions of Evolutionary Algorithms. It is known for its fast convergence, user-friendly nature, and ease of implementation. Additionally, the same set of parameters, such as Population size (NP), Crossover rate (Cr), and Scaling Factor (F), can be applied to address various optimization problems. The process begins with a given set of solutions. Then, a modified or mutant solution is produced for each solution vector in the current set by adding the weighted difference between two candidate solutions to other candidate solutions. This method, known as Differential Evolution (DE), has proven effective and widely applied in various optimization problems in different scientific and engineering domains^59^.

The structure and primary search operators utilized by the DE algorithm are explained as the following:

#### Mutation

In every epoch (t), a mutation operator is applied by DE to generate a new donor vector, also known as a mutant vector, for each target solution. The mutation operator randomly selects three candidate solutions according to Eq. (1); it demonstrates that the donor vector is created by scaling the difference vector between two vectors and then adding the result to the third solution^30^.

1 $${V}_{i,G+1}={x}_{r1,G}+F\left({x}_{r2,G}-{x}_{r3,G}\right)$$

In this process, three distinct integers $r1, r2 and r3$ are randomly selected, and ∈ [1, NP] where NP is a positive integer greater than or equal four. Additionally, these integers are different from the running index i. The differential amplification $\left({x}_{r2,G}-{x}_{r3,G}\right)$ is then amplified by a constant factor F, which ranges from 0 to 2.

#### Crossover

After mutation, a crossover search operator produces an offspring (trial) vector from the target solution. The exponential and binomial crossover search operators are the most frequently used and uncomplicated ones. Please keep in mind that for each decision variable (DV) $j$ in the scenario where ($rand\le {C}_{r}$), do the following:

2 $${u}_{i,j,G}=\left\{\begin{array}{c}{u}_{i,j,G}\,\,\,\,\,\,\,\,\, if \,rand \left(j\right)\le {C}_{r} \,or \,j={j}_{rand} \\ { x}_{i,j,G}\,\,\,\, otherwis{e}{\prime}\,\,\, j=\text{1,2},\dots D \end{array}\right.$$

where a random value ${j}_{rand}$ is selected from the range of, where ${N}_{x}$ is a specified value, a value chosen at random and referred to as “jth evaluation,” denoted by $rand (j)$ is selected from a uniform random number range of [0, 1]. This ensures that at least one DV (design variable) is obtained from the trial vector. The crossover rate ${C}_{r}$, which is used to control the number of variables, is obtained from the donor vector, and it is guaranteed that ${V}_{i,G+1}$ provides at least one parameter to ${u}_{i,j,G}$

#### Selection

A selection operator is utilized to determine the optimal solution by comparing the objective function values of both the parent and offspring. If the offspring has a lower objective function value, it is preserved for the subsequent iterations. If not, the parent vector is mathematically represented within that particular generation, and it is obtained using:

3 $${x}_{i,G+1}=\left\{\begin{array}{c}{u}_{i,G} \,\,\,\,\,\,\,\,\,\,\,\,\,\,\,\,\,\,\,\,\,\,if\left(f\left({u}_{i,G}\right)\le \left({x}_{i,G}\right)\right) \\ { x}_{i,G} \,\,\,\,\,\,\,\,\,\,\,\,\,\,\,\,\,\,\,\,\,\,\,\,\,\,\,\,\,\,\,\,\,\,\,\,\,\,\,\,\,\,\,otherwise \end{array}\right.$$

To determine if it should join generation *G* + *1*, the trial vector ${x}_{i,G+1}$ is evaluated against the target vector ${x}_{i,G}$ using the greedy criterion. If the trial vector ${x}_{i,G+1}$ results in a lower cost function value compared to the target vector ${x}_{i,G}$, then the trial vector ${x}_{i,G+1}$ replaces the target vector ${u}_{i,G}$; if not, the original target vector ${x}_{i,G}$ value is kept.

### The sailfish optimizer (SFO)

Shadravan et al.^22^ developed a unique algorithm called sailfish optimizer (SFO) in 2019, which is based on swarm intelligence and is a population-based algorithm. To devise this technique, the scientists took cues from a pack of predatory sailfish. The approach involves the use of two distinct populations. The sailfish population is responsible for intensifying the search around the current best solution, while the sardine population diversifies the search space. The sailfishes are considered potential solutions, and their positions in the search space represent the problem's variables. The algorithm aims to randomize all search agents’ movement (sailfish and sardine) to the greatest extent possible. Sailfishes are dispersed throughout the search space, while the positions of sardines aid in discovering the optimal solution in the search space.

The algorithm identifies the sardine with the best fitness value as the ‘injured’ fish, with its position denoted as (${P}_{srdinj}^{i}$) at the ${i}^{th}$ iteration. During each iteration, the positions of both sardines and sailfishes are updated. For the ${i}^{th}$ iteration, the position of a sailfish is updated using the ‘elite’ sailfish ${P}_{Slfbest}^{i}$ and the ‘injured’ sardine based on a specific criterion.

The position of sailfishes and sardines is modified at each iteration denoted by $i+$, and the (elite) and (injured) alter or update the position of a sailfish to a new one denoted by. The updating is done according to Eq. (4) ^37^:

4 $${P}_{Slf}^{i+1}={P}_{Slfbest}^{i}-{\mu }_{i}\left(rand*\frac{{P}_{Slfbest}^{i}+{P}_{srdinj}^{i}}{2}- {P}_{Slf}^{i}\right)$$

where the value of $rnd \in (\text{0,1})$ is a random value, and the coefficient ${\mu }_{i}$ is generated by Eq. (5):

5 $${\mu }_{i}=\left(3*rand*PrD-PrD\right)$$

where In each iteration, the prey density ($PrD$), which represents the number of prey available, is determined using Eq. (3). As the number of prey decreases during group hunting, the value of $PrD$ decreases accordingly.

6 $$PrD=1-\frac{{N}_{Slf}}{{N}_{Slf}-{N}_{srd}}$$

Sailfish’s and sardine numbers are represented by ${N}_{Slf} and {N}_{srd,}$ respectively. The ${Num}_{Slf}$ can be calculated according to Eq. (7):

7 $${N}_{Slf}= {N}_{srd}*Prcent$$

Please keep in mind that ($Prcent$) refers to the percentage of the sardine population that constitutes the initial sailfish population. It is also assumed that the initial number of sardines exceeds the number of sailfish.

The positions of the sardines are updated in each iteration according to Eq. (8):

8 $${P}_{Srd}^{i+1}=rand*({P}_{Slfbest}^{i}-{P}_{Srd}^{i}+ATK)$$

The old position and the updated position of the sardine are represented by ${P}_{Srd}^{i}$ and ${P}_{Srd}^{i+1,}$ respectively. While the $ATK$ represents the power of the sailfish attack at each iteration ${i}^{th}$ and can be calculated by Eq. (9):

9 $$ATK=A*\left(1-\left(2*itr*k\right)\right)$$

ATK is crucial in determining the number of sardines that update their positions and the extent of their displacement. Decreasing ATK can facilitate the convergence of search agents. Based on the $ATK$ parameter, the values of $\gamma$ (number of sardines that update their position) and $\delta$(number of variables) of the sardines are computed using Eqs. (10) and (11):

10 $$\gamma =ATK*{N}_{Srd}$$

11 $$\delta =ATK*v$$

where ${N}_{Srd}$ and $v$ denote the sardine number and the number of variables, respectively, if a sardine surpasses the fitness level of any sailfish, the sailfish will adjust its position to follow that sardine. In contrast, the sardine is removed from its population.

To explore the search space effectively, it’s important to select both sailfishes and sardines randomly. Sailfishes have a decreasing attack power after each iteration, allowing sardines to escape from the most aggressive sailfish. This helps to balance the exploration and exploitation of the search space. The $ATK$ parameter is used to find the optimal balance between both of them.

## Methodology of the proposed DESFO

Improving the accuracy of classifiers involves focusing on pertinent features. Some Recent research studies^1,60^ suggest utilizing the methodology of feature selection (FS) to substitute a sizable quantity of insignificant features with a more concise and applicable subset of features. FS categorizes features as essential or non-essential, marking them as 1 or 0. This paper presents a hyped algorithm named (DESFO) which consists of two algorithms, (DE) differential evolution and (SFO) sailfish optimizer, for implementing FS. The algorithm comprises several stages: initialization, position updating, binary conversion, exploration optimization via a new strategy, and exploitation optimization.

Table 2 displays the number of iterations allocated for each algorithm, which is 100. For the proposed algorithm, DESFO, this number was distributed equally between DE and SFO, with 50 iterations each. DE optimized the first 50 iterations to obtain the optimal solution, which was then passed on to SFO to enhance selected relevant features and achieve the best classification accuracy. The following sections provide detailed explanations of each of these stages.

### Initial population generation

The first step in using the DESFO algorithm is generating an initial population of X positions representing potential solutions in a *D*-dimensional space. The population size is determined using a specific formula.

12 $$X=Round\left(10+2*\sqrt{D}\right).$$

X signifies the overall number of positions, while D represents the problem's dimensionality. The position matrix is defined as:

$$M=\left[\begin{array}{c}{m}_{\text{1,1}}, {m}_{\text{1,2}}, \dots {m}_{1,p}\\ {m}_{\text{2,1}}, {m}_{\text{2,2}},\dots {m}_{2,p}\\ \vdots \,\,\,\,\,\,\,\,\,\vdots \,\,\,\,\,\,\,\ddots \,\,\,\,\,\vdots \\ {m}_{X,1}, {m}_{X,2}, \dots {m}_{X,p}\end{array}\right]$$

The ${j}^{th}$ solution is represented by ${M}_{i,j}$, where *j* is the ${j}^{th}$ component. $M$, the initial population, is generated within predefined bounders as:

13 $${M}_{i}^{u}=u\left(\text{0,1}\right)*\left(UB-LB\right)+LB$$

### Position update in DESFO

Updating the position involves using the equations of DE and SFO as described in subsections 3.1 and 3.2. After updating the position, it goes through binary conversion, as explained in Subsection 4.3. The fitness function then assesses the binary-transformed vector to calculate the classification error while keeping the original format of the vector for future updates.

### Position binary conversions

Converting the values of meerkat positions from continuous to binary is necessary before assessing their fitness using the FS method. This is because the DESFO method, which is used to derive the position values, differs from the binary framework of FS, making it challenging to apply the latter directly to binary/discrete problems.

The feature selection (FS) method uses a vector of binary values, where the selected features are represented by 1s, indicating 0s represent their continuous values and the non-selected features. The length of the solution vector is equivalent to the count of features in the original dataset.

A transfer function (TF) has been utilized in the proposed algorithm, which Fang et al. suggested^61^, which has a V-shaped curve and is known for its exceptional global search capability. The function is expressed as follows:

14 $$v\left(y\right)=\alpha *\frac{\text{arctan}\left(y\right)*\frac{\pi }{\sqrt{1+{y}^{2}}}}{\pi }$$

The position value obtained is represented by $y$, and a DESFO position is considered to have a valid TF output where $\alpha$ is less than 0.64 and falls within the range of [0, 1]. The defined update rule for DESFO’s position is based on the following equation:

15 $${Y}_{i}^{bin}=\left\{\begin{array}{c}1, \,\,\,\,\,\,\,\,\,\,\,\,\,\,\,\,\,\,\,If \,rand<v({Y}_{i})\\ 0, \,\,\,\,\,\,\,\,\,\,\,\,\,\,\,\,\,\,\,\,\,\,\,\,\,\,\,\,\,\,\,\,\,\,otherwise\end{array}\right.$$

### Fitness evaluation

The DESFO framework and a new FS-based technique incorporate k-NN and RF as evaluative mechanisms. The k-NN method^62^ selects the most common class among the closest neighbors to predict the classification of new instances. On the other hand, the RF, explained in^44^, uses decision trees to recursively divide the training data into small sets, which helps optimize the classification task by using an impurity criterion such as information gain or “gini” index^63^. These classifiers are particularly efficient in handling high-dimensional data and require minimal computational effort, as stated in^62^.

Achieving the right balance between accuracy and feature set size is crucial in DESFO. While opting for a smaller feature set can improve the precision of classifiers such as k-NN and RF, it may also compromise accuracy due to the reduced feature set^64^. The relationship between the size of the feature set and the preferred features is inversely proportional, which means there is a potential trade-off between accuracy and feature set size. Therefore, the PMBH method is vital in balancing feature selection and classification accuracy^65^.

When assessing the effectiveness of an algorithm, it is essential to consider the trade-off between precision and feature size. This trade-off can be mathematically represented as:

16 $$FIT={\alpha }_{1}*\left(1-accuracy\right)+{\alpha }_{2}+\left|\frac{\left|{D}^{*}\right|}{\left|D\right|}\right|$$

In the given equation, there are two weight coefficients, α1 and α2, where α1 is a value between 0 and 1, and α2 is determined by subtracting α1 from 1. These values have been determined through extensive testing, as mentioned in reference, and the expression $represents$ the ratio of the selected features to the total number of features in the original dataset. The main objective of this design is to increase precision while reducing the length of the feature set, as suggested in reference^38^. The value |D ∗| represents the size of the selected feature set, while |D| represents the total number of features in the original dataset.

### Improving exploration

**Figure 1:**
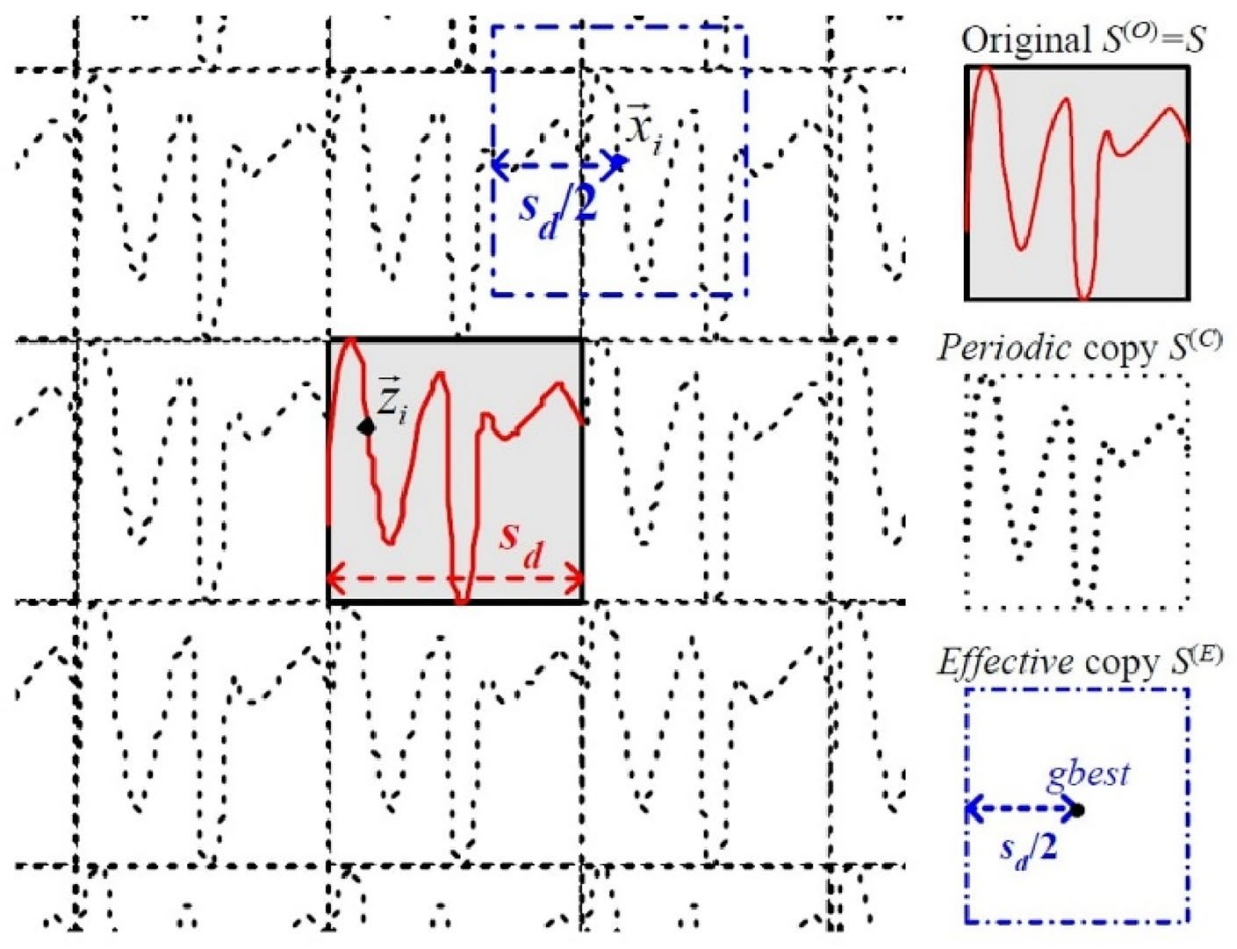
PMBH^70^.

Search agents like meerkats tend to explore outside their assigned search areas to find optimal solutions. However, issues may arise when using boundary-handling techniques to keep an agent within the initial search territory, as discussed in^61^. The two primary traditional methods for boundary handling are Boundary and Random modes. In Boundary mode, if a solution's dimension d goes beyond the search space S, it gets repositioned to the nearest boundary, either lower bound L or upper bound U. Conversely, dimension d of S receives random value mutations in Random mode. These traditional methods, however, have limitations in fully exploring the search space. Therefore, Periodic Mode Boundary Handling (PMBH) was developed as per^61^, aiming to improve the exploration phase. PMBH allows for infinite search space for agent movement, consisting of periodic replicas of the original space S, maintaining the same fitness landscape, as shown in Fig. 1.

### Exploitation optimization

This particular segment notices the updated LS principles of the enhanced DESFO. These principles aim to improve the efficiency of algorithms and ensure better utilization by generating a fresh population with optimal positions while maintaining the essential structure.

Three main principles guide the proposed approach. Firstly, to address the limitation of the original algorithm that lacks a mechanism to recall and preserve the best solutions over iterations, a binary matrix has been introduced to store the top solutions obtained previously. Secondly, repetitive best solution patterns resulting from binary conversion can reduce exploitation effectiveness, which can be improved by incorporating distinct solutions in the binary matrix. Lastly, the LS strategy relies on identifying solutions close to the best discovered by converting continuous positions into binary format and following a constrained normal distribution, as shown in Eq. (17).

17 $${x}_{d}^{l+1}={x}^{L}+{\beta x}^{L}$$

The solution obtained through minor mutation slightly deviates from the current best, due to a random factor represented by $\beta$ which is normally distributed $N(0.0, 0.4)$. The optimal solution is initially added to an empty set to find local search solutions. The set has a fixed maximum size, ${LS}_{max}$. Then, a new solution is generated by applying Eq. (17) on the current ${g}_{best}$, which is then converted to binary and assessed for fitness. If this new solution outperforms the current best, it is considered the best solution.

### The flowchart and *Pseudo* code of DESFO

**Figure 2:**
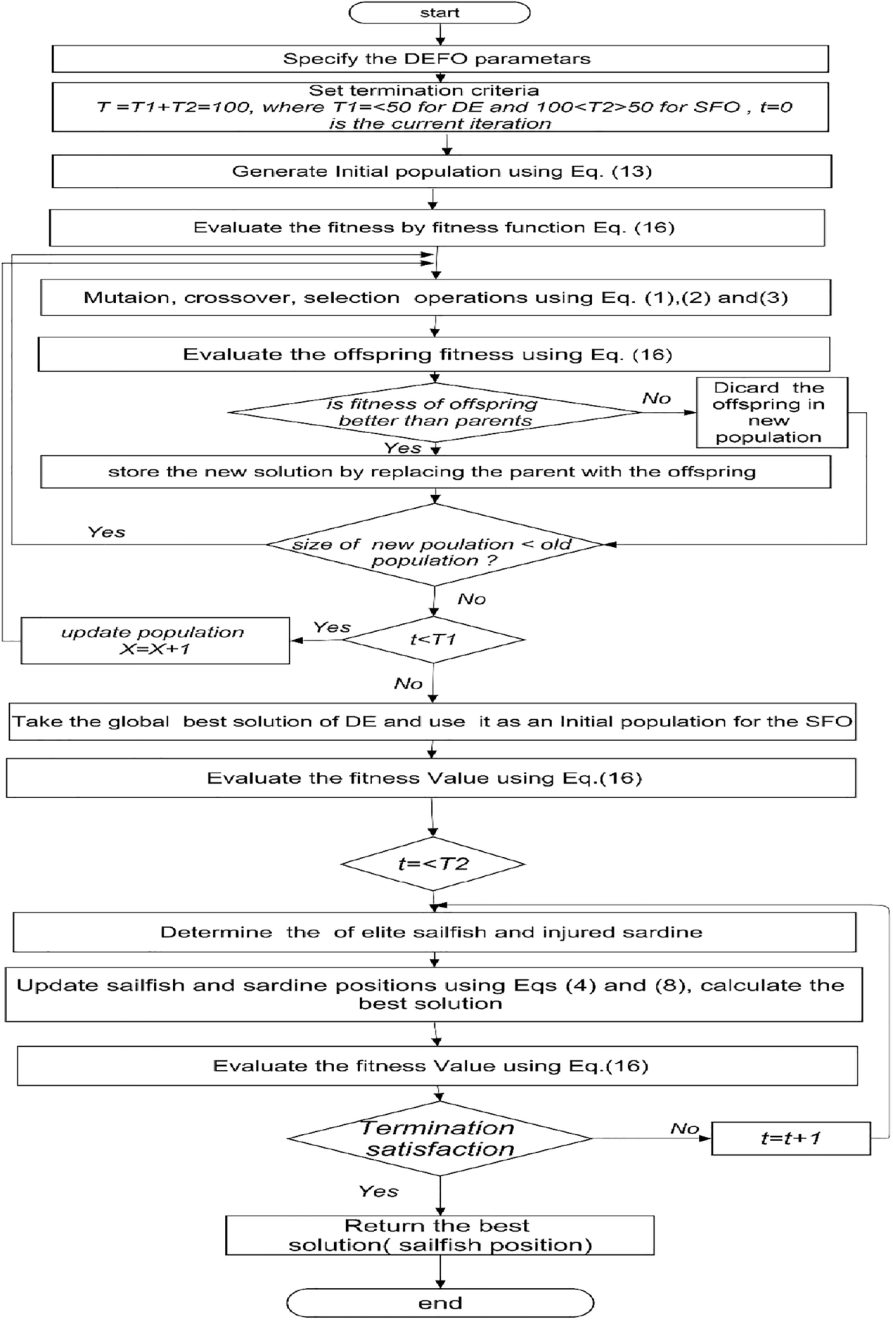
DESFO flowchart.

In Fig. 2, the steps of the proposed DESFO algorithm are demonstrated.

**Algorithm 1:**
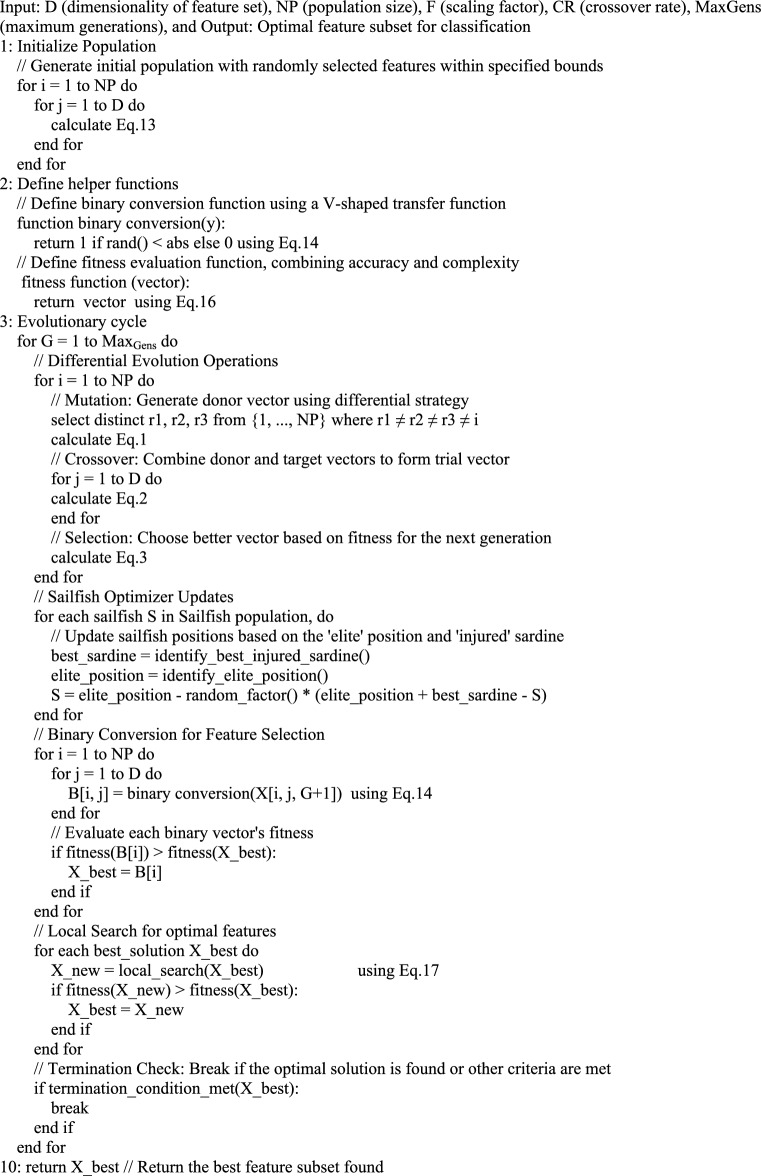
Differential Evolution with Sailfish Optimizer (DESFO)

### Complexity analysis

In analyzing the complexity of the DESFO, we can delve deeper into the computational processes involved. This includes looking at the computational demands of evaluating classifiers and the benefits of using combined methods in terms of efficiency.

- Complexity Breakdown by Component
  1. Initialization: Initializes NP individuals, each possessing D features. This operation has a complexity of *O* (*NP* × *D*).
  2. Differential Evolution Operations:
- Mutation: For the mutation step to be executed across all individuals, it involves choosing three different individuals and then computing the vector differences for each, which amounts to a complexity of (*D*) for each individual. Consequently, the total complexity for the mutation step applied to all individuals is *O* (*NP* × *D*).
- Crossover: for each person, determined by the probability *CR*, this leads to *O* (*NP* × *D*).
- Selection: Evaluating and selecting the better individual between the target and trial vector typically involves fitness computation, which can be a significant factor depending on the complexity of the fitness function. the complexity is *O*(*NP*)
  3- Sailfish Optimizer Updates:
- Position Update: Each sailfish updates its position based on the positions of elite and injured sardines, the complexity per generation is *O*(*NP*)
  4- Binary Conversion and Fitness Evaluation:
- Binary Conversion: Each of the *D*D features of each *NP*NP individuals is converted from a real number to a binary value based on a transfer function, totaling *O*(*NP* × *D*)
- Fitness Evaluation: The evaluation of fitness depends on the classification algorithm used. For k-NN or RF, the time complexity might depend on the number of features *D* and possibly the sample size if a wrapper method is used. The complexity is therefore *O* (*NP* × *f* (*D*))*,* where (*D*) represents the computational complexity of evaluating one individual.
  5- Local Search:
- Local Search Operations: Assuming that local search is applied to a subset of the population (say *k* best individuals) and each local search operation has a complexity *O* ((*D*))*,* where (*D*) might involve multiple evaluations of minor variations of the individual. If *LS* iterations of local search are performed for each individual, the complexity for this part would be (*k* × *LS* × (*D*))*.*
- Overall Complexity

Combining all these elements, the total complexity per generation of the DESFO algorithm would be: (*NP*×*D*+*NP*×*D*+*NP*+*NP*×*D*+*NP*×(*D*)+*k*×*LS*×*g*(*D*))

This simplifies to: (3×*NP*×*D*+*NP*×(*D*)+*k*×*LS*×*g*(*D*))

For all generations, *MaxGens*, the overall complexity becomes: (*MaxGens*×(3×*NP*×*D*+*NP*×*f*(*D*)+*k*×*LS*×*g*(*D*)))

- Comparing the complexity of DESFO with DE and SFO shows that the total complexity for DESFO is *O*(*MaxGens* × (3 × *NP* × *D* + *NP* × *f*(*D*) + *k* × *LS* × *g*(*D*))) however SFO complexity is *O(MaxGens* × *4* × *NP*_*)*_ and DE complexity is *O(MaxGens* × *3* × *N*_*P*_*)which mean that The* DESFO has more computational complexity due to its integrated steps and phases

## Experimental results and analysis

The following part of the paper presents the results from the proposed DESFO algorithm and compares them with those reported in prior studies. To verify the proposed algorithm, 14 multi-scale benchmarks were utilized—the mean values in the results are represented as evaluation metrics. To showcase the efficacy of the suggested algorithm, in all experiments, we employed the datasets that are elaborated in subsection 5.1, Moreover, the metaheuristic techniques’ main parameters utilized in this paper are outlined in subsection 5.2, in subsection 5.3, evaluation measures are explained, then, in subsection 5.4, the proposed DESFO algorithm is evaluated and compared with the k-NN and RF algorithms to investigate their respective results, in subsection 5.5, An investigation was conducted to compare the outcomes of the suggested DESFO algorithm with those of other methods, Convergence graphs are depicted in Sect. 5.6, in subsection 5.7, the Wilcoxon's test is conducted to assess the credibility of differences in fitness rates between the proposed DESFO algorithm and its counterparts and the final Sect. 5.8 is for discussion of the results.

### Benchmarks description

The proposed algorithm’s performance is demonstrated using 14 multi-domain features and instance benchmarks. These benchmarks are obtained from the UCI machine learning repository^57^. A variety of attributes and instances in each benchmark is beneficial in validating the proposed algorithm. Table 1 provides an overview of the benchmarks used in this paper, along with their respective properties and descriptions. The datasets shown in Table 1 are sorted in descending according to the number of features.

**Table 1:** Dataset characteristics.

#	Benchmarks	#. features	#. instances	#.classes
1	PenglungEW	325	73	7
2	IonosphereEW	34	351	2
3	SonarEW	60	208	2
4	WaveformEW	40	5000	3
5	KrVsKpEW	36	3196	2
6	BreastEW	30	569	2
7	Lymphography	18	148	4
8	Vote	16	300	2
9	Zoo	16	101	7
10	Exactly2	13	1000	2
11	M-of-n	13	1000	2
12	WineEW	13	178	3
13	Breast cancer	9	699	2
14	Tic-tac-toe	9	958	2

### Parameters configuration

The DESFO algorithm proposed in this study was evaluated against several meta-heuristic algorithms, including the two original algorithms that were combined, the Differential Evolution (DE) algorithm^30^ and the sailfish optimization (SFO) algorithm^22^, as well as nine of the other algorithms, including Harris Hawks Optimization (HHO)^66^, Particle Swarm Optimization (PSO)^67^, Bat Algorithm (BA)^17^, Whale Optimization Algorithm (WOA)^68^, Grasshopper Optimization Algorithm(GOA)^69^, Grey Wolf Optimization (GWO)^18^, Bird Swarm Algorithm (BSA)^70^, Henry gas solubility optimization (HGSO)^71^, and Artificial Bee Colony (ABC)^11^. In this work, the ML classifiers' primary parameters have been established as follows: the k-NN classifier’s Euclidean distance metric has been approximated to be 5. The estimation was based on the outcomes obtained from previous papers, such as^72^. On the other hand, the Random forest (RF) classifier^73^ is a popular machine-learning algorithm often used for complex tasks such as time-series forecasting, image classification, facial expression recognition, action recognition and detection, visual tracking, label distribution learning, and more. Every method is evaluated on each dataset by conducting 30 distinct experiments. The results are reported according to the mean performance measures. To maintain equality in the evaluation process, each method had a population size of 10 and a maximum of 100 iterations. The size of the datasets used was proportional to the complexity of the problem. The exploration of the continuous search space was confined yet extensive by establishing the search domain as [−1, 1].

A validation process is necessary to assess the optimality achieved by the outcomes in the framework, so a tenfold cross-validation method is employed. This ensures that the values obtained are reliable. The benchmark is randomly split into two subsets, with 80% of the benchmark used for training and the remaining for testing purposes^3^. During the learning process of the machine learning classifier, sunset for training is used and optimized, while the test subset is used to evaluate the selected features. Table 2 displays the standard configurations for all techniques and the parameter settings for each method, which were determined based on the original variants and the data included in their initial publications. Python is used to run the processes on a computer system environment equipped with a CPU, an Intel i7 processor, RAM, which is 16 GB, and a GPU, which is NVIDIA GTX 1050i.

**Table 2:** All Algorithms parameter’s configuration.

#	Algorithm	Parameters configuration
1	All Algorithms	#of Runs = 30 #*T* denoted Iterations = 100 *N* is the size of the population = 10 *D* = *#* of Attributes in the Used benchmarks
2	DE	F is an amplifier constant ∈ [0, 2], Cr is the crossover rate, Rand ∈ [0, 1] j_rand_ ∈ [1,2.Nx] r1, r2, r3 ∈ [1, NP], NP ≥ 4
3	SFO	*pp* = 0.1 is the in-between ratio of sardines and sailfish ε = 0.0001 A = 1
4	ABC	# bees = 16, # scout bees = 3, # onlooker bees = 4
5	PSO	Inertia weight (ωmax = 0.9ωmin = 0.4), Acceleration parameter (c2 = c1 = 1.2)
6	BA	Loudness A = 0.8 Lower and upper pulse frequencies = 0, 10 Pulse emission rate r = 0.95
7	GWO	a is reduced from 2 to 0 linearly
8	WOA	a is reduced from 2 to 0 b = 1.0 *p* = 0.5 linearly
9	GOA	Cmax = 1 and Cmin = 0.00004
10	HHO	E ∈ [− 1, 1] denotes Rabbit energy
11	BSA	*ff* = 10 and denote the flight frequent *fl* = 0.5 is the Following coefficient (a1 = a2 = 1.0) are the Effects on the vigilance behaviors of birds (c1 = c2 = 1.5) are two coefficients of the Acceleration p = 0.8 is food foraging Probability
12	HGSO	# of clusters = 2 l_1_ = 5E − 03, l_2_ = 1E + 02, and l_3_ = 1E − 02 *α* = *β* = 0.1 and K = 1.0

### Metrics of performance

The DESFO algorithm performance is compared to other methods, and each approach is assessed independently in 30 runs per benchmark. The evaluation of the FS strategy employs certain measures to conduct this assessment.

**Mean accuracy:** The accurate data classification rate (${Mean}_{acc}$) can be determined by executing the method independently for 30 runs:

18 $${Mean}_{acc}=\frac{1}{30}\frac{1}{m} \sum_{k=1}^{30}\sum_{r=1}^{m}match({PL}_{r},{AL}_{r})$$

where mean accuracy is represented by ${Mean}_{acc}$, while the number of samples in the subset of testing is denoted by *m*, the predicted class label for a sample is denoted by *PLr*. In contrast, the reference class label is denoted by *ALr*. A function called match (*PL*_*r*_*, AL*_*r*_) compares these labels. When *PLr* is equal to *ALr*, the value of match (*PLr*, *ALr*) is 1; otherwise, it is 0.

**Mean fitness value**: The metric (Mean_*Fit*_) measures the average fitness results achieved through the recommended approach by running it individually for 30 runs. This highlights how decreasing the number of chosen features can lead to a lower error classification rate, as per Eq. (16). The best result is indicated by the minimum value, which is evaluated based on fitness as:

19 $${Mean}_{Fit}=\frac{1}{30}\sum_{k=1}^{30}{f}_{*}^{k},$$

The ${Mean}_{Fit}$ denotes the mean or average fitness value, while ${f}_{*}^{k}$ indicates the best possible fitness outcome attained during each run of the 30 k-th runs.

**The mean number of features selected**: This metric, which *MeanFeat* denotes, represents the mean or average count of chosen features obtained by performing the technique independently for 30 runs and is defined as:

20 $${Mean}_{Feat}=\frac{1}{30}\sum_{k=1}^{30}\frac{\left|{d}_{*}^{k}\right|}{\left|D\right|},$$

where $\left|{d}_{*}^{k}\right|$ denotes the selected features, the number of features for the optimal solution for each run of the thirty k-th runs, while |D| denotes the number of the complete features used from the benchmarks.

- **Wilcoxon’s rank-sum test:** To gain a deeper insight into the importance of the method discussed statistical evidence must demonstrate its effectiveness. Therefore, the efficacy of the results derived from the methods used is often validated by employing the Wilcoxon rank-sum non-parametric test. This is favored for its ability to statistically distinguish the significance and dependability of various competing methods^74^. In this study, the focus is on evaluating the proposed DESFO method in comparison with other algorithms. A null hypothesis is put forward, suggesting no difference in performance between the DESFO algorithm and the others when compared pairwise. Conversely, if proven otherwise, the DESFO algorithm outperforms the rest. The assessment hinges on the calculation of a p-value through the Wilcoxon rank-sum test, which helps analyze the differences in outcomes from 30 separate executions of both the DESFO and competing algorithms.

### *The results of ML *classifiers* (k-NN and RF) and DESFO*

The mean accuracy (${Mean}_{acc}$) was used to compare the performance of the presented ML classifiers (RF and k-NN) with the proposed methods (DESFO-RF and DESFO-K-NN) and the mean number of selected features (${Mean}_{Feat}$) in this subsection are also given. This was done to evaluate the effectiveness and scope of the DESFO approach.

#### Comparisons of DESFO- K-NN and K-NN

In Table 3, a comparison between the DESFO-*K-NN* technique and the basic *K-NN* algorithm is demonstrated. The evaluation is centered on two metrics to measure performance: the average accuracy of classification (*Mean*_*Acc*_) and the average count of selected features (*Mean*_*Feat*_).

**Table 3:** Comparison of Maean_*Acc*_ and Mean_*Feat*_ for DESFO-K-NN & the basic K-NN.

Benchmarks	Accuracy			Features			
	*Basic K-NN*	DESFO- *K-NN*(Mean)	Increasing (%)	*Basic K-NN*	DESFO- K- NN(Mean)	Decreasing (%)	
PenglungEW	0.5333	**0.6533**	22.50	325	**108.2**	66.71	
IonosphereEW	0.8451	**0.9324**	10.33	34.0	**9.700**	71.47	
SonarEW	0.8571	**0.9857**	15.00	60.0	**23.60**	60.67	
WaveformEW	0.804	**0.8480**	5.470	40.0	**23.20**	42.00	
KrVsKpEW	0.9656	**0.9822**	1.720	36.0	**20.10**	44.17	
BreastEW	0.9211	**0.9649**	4.760	30.0	**6.300**	79.00	
Lymphography	0.7000	**0.8400**	20.00	18.0	**8.500**	52.78	
Vote	0.9333	**1.0000**	7.150	16.0	**4.100**	74.38	
Zoo	0.9048	**1.0000**	10.52	16.0	**5.200**	67.50	
Exactly2	0.7400	**0.7935**	7.230	13.0	**6.300**	51.54	
M-of-n	0.8800	**1.0000**	13.64	13.0	**6.100**	53.08	
WineEW	0.5833	**1.0000**	71.44	13.0	**4.000**	69.23	
BreastCancer	0.6214	**0.9857**	58.63	9.00	**6.000**	33.33	
Tic tac toe	0.8441	**0.8542**	1.200	**9.00**	**9.000**	0.00	
W Score T L	0	**14**	*	0	**13**	*	
	0	**0**		1	**1**		
	14	**0**		13	**0**		

Superior values are in [bold].

After analyzing Table 3, it is worth mentioning that the DESFO–K-NN technique led to an increase in Mean_*Acc*_ on all benchmarks. The increase was more than 15% on four of them. Moreover, Mean_*Acc*_ had a score of over 93% on nine out of the total fourteen benchmarks. It even achieved 100% Mean_*Acc*_ on four of them. It is worth mentioning that the Mean_*Feat*_ has decreased in 93% of the benchmarks due to implementing the DESFO–K-NN method as suggested. However, the DESFO–K-NN method could not improve the Mean_*Feat*_ on the Tic-tac-toe benchmark. Finally, it was found that the DESFO–K-NN technique outperformed the basic K-NN in terms of Mean_*Acc*_ and most of the benchmarks. On the other hand, the suggested MeanFeat of the DESFO–k-NN approach has shown promising results in feature selection compared to the basic k-NN tested with the chosen datasets.

#### Comparisons of DESFO- RF and RF

In Table 4, a comparison between the DESFO-*RF* algorithm and the basic *RF* algorithm is demonstrated. The comparison is based on two performance metrics: the mean accuracy of classification (Mean_*Acc*_) and the mean number of chosen features (Mean_*Feat*_).

**Table 4:** Comparison of Maean_*Acc*_ and Mean_*Feat*_ for DESFO-RF & the basic RF.

Benchmarks	Accuracy			Features		
	*Basic RF*	DESFO- *RF*(Mean)	Increasing (%)	*Basic RF*	DESFO- *RF*(Mean)	Decreasing (%)
PenglungEW	0.3333	**0.7667**	130.03	325	**155.6**	52.12
IonosphereEW	0.9014	**0.9732**	7.9700	34.0	**014.6**	57.06
SonarEW	0.7857	**0.9286**	18.190	60.0	**027.6**	54.00
WaveformEW	0.7690	**0.8197**	6.5900	40.0	**020.9**	47.75
KrVsKpEW	0.7953	**0.9487**	19.290	36.0	**017.1**	52.50
BreastEW	0.9298	**0.9947**	6.9800	30.0	**012.2**	59.33
Lymphography	0.7333	**0.8933**	21.820	18.0	**009.1**	49.44
Vote	0.9000	**1.0000**	11.110	16.0	**003.2**	80.00
Zoo	0.9524	**1.0000**	5.0000	16.0	**004.5**	71.88
Exactly2	0.7500	**0.7650**	2.0000	13.0	**005.0**	61.54
M-of-n	0.8000	**0.9950**	24.380	13.0	**006.3**	51.54
WineEW	**1.0000**	**1.0000**	0.0000	13.0	**003.0**	76.92
BreastCancer	0.9643	**0.9857**	2.2200	9.00	**005.1**	43.33
Tic tac toe	0.7500	**0.8698**	15.970	9.00	**007.0**	22.22
W Score T L	0	**13**	*	0	**14**	*
	1	**1**		0	**0**	
	13	**0**		14	**0**	

Superior values are in [bold].

After analyzing Table 4, it is worth mentioning that the DESFO–RF technique led to an increase in Mean_*Acc*_ on 93% of all benchmarks. The increase was more than 15% on four of them. Moreover, Mean_*Acc*_ had a score of over 92% on nine out of the total fourteen benchmarks. It even achieved 100% Mean_*Acc*_ on three of them. It is monitored that DESFO-RF and basic RF are equal in accuracy in one of the WineEW benchmarks. It is worth mentioning that the Mean_*Feat*_ has decreased in 100% of the benchmarks due to implementing the DESFO–RF method as suggested. However, finally, it was found that the DESFO–RF method outperformed the original RF algorithm in terms of Mean_*Acc*_ in most of the benchmarks and Mean_*Feat*_. The suggested DESFO–RF approach has shown promising results in feature selection compared to the main RF on the chosen benchmarks.

### DESFO results versus other MH algorithms

To prove the effectiveness of DESFO in comparison with DESFO-RF and DESFO-K-NN, which rely on *RF* and *k-NN* classifiers, respectively, a comparison was made between DESFO and other meta-heuristic methods such as DE, SFO, ABC, PSO, BA, GWO, WOA, GOA, HHO, BSA, and HGSO, all of which were conducted under identical conditions. The comparison results were measured in terms of mean fitness value (Maean_*Fit*_), mean accuracy (Maean_*Acc*_), and mean number of features selected (Mean_*Feat*_).

#### Comparisons based on the RF classifier

Table 5 presents the fitness values obtained from the proposed DESFO-RF meta-heuristic optimization algorithm, compared with those of other advanced optimization techniques in addressing the FS issue. Table 5 shows that DESFO-RF showed superior performance compared to other methods. In the FS problem, it scored the highest in 8 benchmarks and achieved the same score as the others in 2 benchmarks. This led to a more significant impact in 10 out of the 14 benchmarks, equivalent to 71% of all the benchmarks. Furthermore, the benchmark employed in this research comprises benchmarks of varying sizes, demonstrating the ability of DESFO-RF to deliver consistent performance across the entire range of benchmarks, regardless of their size. It was observed that DESFO–RF missed out on 4 benchmarks, but the results obtained were much closer to the methods used by SFO and ABC when the mean fitness values were compared. This indicates that the DESFO–RF has better outcomes than its competitors. It has been discovered that the DESFO-RF method suggested by the team ranked first in all benchmarks except for SFO. This provides further evidence of the effectiveness of the proposed method over other techniques used by competitors.

**Table 5:** Results comparison of the mean fitness value (Mean_*Fit*_) based on RF classifier for DESFO with other. MH methods

Benchmarks	DESFO	DE	SFO	ABC	PSO	BA	GWO	WOA	GOA	HHO	BSA	HGSO
PenglungEW	**0.2358**	0.2623	0.2423	0.2499	0.3084	0.3152	0.2953	0.2952	0.3085	0.3019	0.2821	0.3357
IonosphereEW	**0.0308**	0.0417	0.0334	0.0381	0.0447	0.0493	0.0417	0.0442	0.0422	0.0457	0.0405	0.0501
SonarEW	**0.0753**	0.1064	0.0849	0.0977	0.1177	0.1467	0.1108	0.0993	0.1013	0.118	0.1017	0.1472
WaveformEW	**0.1837**	0.1967	0.1853	0.1889	0.1991	0.2075	0.1891	0.1965	0.1934	0.1953	0.1949	0.2103
KrVsKpEW	**0.0555**	0.0676	0.0604	0.0622	0.0731	0.0784	0.0619	0.0657	0.0635	0.0688	0.0641	0.0798
BreastEW	**0.0093**	0.0165	0.0115	0.0118	0.0219	0.0195	0.0165	0.0191	0.0192	0.0202	0.0136	0.0278
Lymphography	0.1107	0.1505	**0.1071**	0.128	0.1603	0.1666	0.1373	0.1535	0.1299	0.1435	0.137	0.1903
Vote	0.0020	0.0032	**0.0018**	0.0023	0.0037	0.0144	0.0027	0.0026	0.0026	0.0023	0.0027	0.0123
Zoo	**0.0028**	0.0033	0.0029	0.0032	0.0035	0.004	0.0033	0.0036	0.0034	0.0034	0.0035	0.0047
Exactly2	**0.2365**	0.242	0.2385	0.241	0.2462	0.2466	0.241	0.2447	0.2431	0.2457	0.238	0.2488
M-of-n	0.0098	0.0556	**0.0078**	0.0184	0.0732	0.0751	0.013	0.028	0.0332	0.0255	0.0367	0.0839
WineEW	**0.0023**	0.0027	**0.0023**	0.0027	0.0028	0.0035	0.0026	0.0025	0.0028	0.0029	0.0026	0.0044
BreastCancer	0.0192	0.0194	**0.0191**	**0.0191**	0.0198	0.0218	0.0197	0.0196	0.0196	0.0201	0.0194	0.0200
Tic-tac-toe	**0.1367**	0.1375	**0.1367**	**0.1367**	0.1383	0.1391	**0.1367**	0.1383	0.1375	0.1403	**0.1367**	0.1482
W Score T L	**8**	0	3	0	0	0	0	0	0	0	0	0
	**2**	0	3	2	0	0	1	0	0	0	1	0
	**4**	14	8	12	14	14	13	14	14	14	13	14

Superior values are in [bold].

Table 6 compares the classification accuracy means of the presented DESFO-RF with other advanced metaheuristic optimization algorithms in tackling the FS issue, as per the empirical findings. It’s worth mentioning that, according to Table 6, the DESFO-RF approach showed better performance than all other methods in terms of accuracy mean across seven benchmarks. Moreover, it delivered equivalent results to other methods across five benchmarks but needed to be more fortunate to outperform them in two benchmarks. However, the DESFO-RF approach was significantly more effective than other methods in 12 out of 14 benchmarks, equivalent to 85.7% of all the benchmarks. Also, it's worth noting that the SFO method was ranked second on several benchmarks. It showed a slight improvement of 0.0034% on the Lymphography benchmark and 0.0020% on the M-of-n benchmark while achieving the same score as the top performer on five other benchmarks.

**Table 6:** Results comparison of the mean accuracy (Mean_*Acc*_) based on RF classifier for DESFO with other MH methods.

Benchmarks	DESFO	DE	SFO	ABC	PSO	BA	GWO	WOA	GOA	HHO	BSA	HGSO
PenglungEW	**0.7667**	0.74	0.76	0.7533	0.6933	0.6867	0.7067	0.7067	0.6933	0.7	0.72	0.6667
IonosphereEW	**0.9732**	0.962	0.9704	0.9662	0.9592	0.9549	0.962	0.9592	0.962	0.9577	0.9634	0.9549
SonarEW	**0.9286**	0.8976	0.919	0.9071	0.8857	0.8571	0.8929	0.9048	0.9024	0.8857	0.9024	0.8571
WaveformEW	**0.8197**	0.8062	0.8179	0.8145	0.8039	0.7955	0.814	0.8070	0.8101	0.8076	0.8075	0.7937
KrVsKpEW	**0.9487**	0.9367	0.9441	0.9423	0.9313	0.9264	0.942	0.9384	0.9406	0.9350	0.9403	0.9252
BreastEW	**0.9947**	0.9877	0.993	0.993	0.9825	0.9851	0.9877	0.9851	0.9851	0.9842	0.9912	0.9772
Lymphography	0.8933	0.8533	**0.8967**	0.8767	0.8433	0.8367	0.8667	0.8500	0.8733	0.8600	0.8667	0.8133
Vote	**1.0000**	**1.0000**	**1.0000**	**1.0000**	**1.0000**	0.9883	**1.0000**	**1.0000**	**1.0000**	**1.000**	**1.0000**	0.9917
Zoo	**1.0000**	**1.0000**	**1.0000**	**1.0000**	**1.0000**	**1.0000**	**1.0000**	**1.0000**	**1.0000**	**1.0000**	**1.0000**	**1.000**
Exactly2	**0.7650**	0.7595	0.7630	0.7605	0.7555	0.755	0.7605	0.757	0.7585	0.7555	0.7635	0.7515
M-of-n	0.9950	0.9495	**0.9970**	0.9865	0.9325	0.93	0.992	0.977	0.972	0.9795	0.9685	0.9220
WineEW	**1.0000**	**1.0000**	**1.0000**	**1.0000**	**1.0000**	**1.0000**	**1.0000**	**1.0000**	**1.0000**	**1.0000**	**1.0000**	**1.0000**
BreastCancer	**0.9857**	**0.9857**	**0.9857**	**0.9857**	**0.9857**	0.9836	**0.9857**	**0.9857**	**0.9857**	**0.9857**	**0.9857**	**0.9857**
Tic-tac-toe	**0.8698**	0.8688	**0.8698**	**0.8698**	0.8677	0.8667	**0.8698**	0.8677	0.8688	0.8656	**0.8698**	0.8573
W Score T L	**7**	0	2	0	0	0	0	0	0	0	0	0
	**5**	4	5	5	4	2	5	4	4	4	5	3
	**2**	10	7	9	10	12	9	10	10	10	9	11

Superior values are in [bold].

Table 7 compares the mean number of selected features between the DESFO-RF method and other popular meta-heuristic optimization algorithms commonly used for feature selection (FS) strategy. When Table 7 is analyzed, the observation shows that DESFO-RF and SFO produce similar results regarding the number of selected features, and both outperform the other algorithms. These two techniques won in two benchmarks and tied in three benchmarks, surpassing the other algorithms: DE, ABC, PSO, BA, GWO, WOA, GOA, HHO, BSA, and HGSO. However, it is important to note that this does not necessarily imply a tie in classification accuracy between DESFO and SFO. DESFO has demonstrated superiority over other algorithms. Furthermore, it should be kept in mind that choosing the smallest number of characteristics may negatively impact classification accuracy.

**Table 7:** Results comparison of the mean number of features selected (Mean_*Feat*_) based on the RF classifier for DESFO with other MH methods.

Benchmarks	DESFO	DE	SFO	ABC	PSO	BA	GWO	WOA	GOA	HHO	BSA	HGSO
PenglungEW	155.6	160.7	**153.3**	183.7	156.3	161.1	159.9	155	158.8	160.2	160.2	184.7
IonosphereEW	14.60	13.90	14.00	15.8	14.6	16.00	13.90	**12.9**	15.50	13.10	14.30	18.70
SonarEW	**27.60**	30.20	28.70	34.7	**27.6**	31.50	28.30	29.9	28.20	29.30	30.10	34.90
WaveformEW	20.90	19.30	20.10	21.1	20.0	20.20	19.80	21.7	21.70	19.10	**17.50**	24.20
KrVsKpEW	17.10	18.00	18.00	18.3	18.2	19.90	16.40	17.2	17.10	**15.90**	18.20	20.60
BreastEW	**12.20**	12.90	13.80	14.7	13.5	14.20	12.90	13.0	13.40	13.60	14.60	15.70
Lymphography	9.100	9.500	8.600	10.6	9.40	8.800	9.500	9.00	**8.100**	8.900	9.000	9.900
Vote	3.200	5.100	**2.800**	3.60	5.90	4.600	4.300	4.20	4.100	3.600	4.300	6.400
Zoo	**4.500**	5.300	4.600	5.10	5.60	6.400	5.300	5.70	5.400	5.500	5.600	7.500
Exactly2	5.000	5.100	5.000	5.00	5.40	5.200	5.000	5.40	5.200	4.800	5.000	**3.600**
M-of-n	**6.300**	7.300	**6.300**	6.50	8.30	7.500	6.600	6.80	7.100	6.800	7.200	8.700
WineEW	**3.000**	3.500	**3.000**	3.50	3.70	4.500	3.400	3.30	3.600	3.800	3.400	5.700
BreastCancer	5.100	5.300	**5.000**	**5.00**	5.70	5.500	5.600	5.50	5.500	6.000	5.300	5.900
Tic-tac-toe	7.000	6.800	7.000	7.00	6.60	6.400	7.000	6.60	6.800	6.500	7.000	**6.200**
W Score T L	**2**	0	**2**	0	0	0	0	0	0	0	0	2
	**3**	0	**3**	1	1	0	0	1	1	1	1	0
	**9**	14	**9**	13	13	14	14	13	13	13	13	12

Superior values are in [bold].

#### Comparisons based on the K-NN classifier

Table 8 compares the average fitness values between the proposed DESFO-K-NN and other advanced MH optimization algorithms in addressing the FS problem. After examining Table 8, the DESFO-K-NN outperformed all other methods in 9 benchmarks and tied in 2 benchmarks in the FS problem. This indicates that DESFO-K-NN had a significantly better impact on 11 out of 14 benchmarks, accounting for 85.7% of all benchmarks. Additionally, the study employed a benchmark of both large and small-scale benchmarks, indicating that DESFO-K-NN can deliver consistent performance across the entire range of benchmarks, irrespective of their size. For the two missing benchmarks, it has been noted that DESFO-K-NN has produced almost equivalent outcomes to other techniques in terms of mean fitness values. This highlights the superior results of DESFO-K-NN. Except for SFO in two benchmarks (vote and zoo), none of the competing methods are ranked first compared to DESFO-K-NN. Hence, it is evident that DESFO-K-NN is superior to the suggested competitor’s methods. In addition, the results of the comparison between DESFO-K-NN and other metaheuristic optimization algorithms in terms of classification accuracy values for feature selection strategy are presented in Table 9. The table shows the empirical outcomes of this comparison.

**Table 8:** Results comparison of the mean fitness value (Mean_*Fit*_) based on the K-NN classifier for DESFO with other MH methods.

Benchmarks	DESFO	DE	SFO	ABC	PSO	BA	GWO	WOA	GOA	HHO	BSA	HGSO
PenglungEW	**0.3465**	0.394	0.3544	0.3742	0.3941	0.3943	0.3742	0.3808	0.3875	0.3803	0.3939	0.4011
IonosphereEW	**0.0698**	0.0888	0.0784	0.0832	0.0958	0.1088	0.0873	0.0846	0.0848	0.0743	0.0872	0.1112
SonarEW	**0.0181**	0.0377	0.0206	0.0258	0.0357	0.0607	0.028	0.035	0.0305	0.0249	0.0277	0.0525
WaveformEW	**0.1563**	0.1734	0.1629	0.1642	0.1771	0.178	0.1657	0.17	0.1682	0.1626	0.1684	0.1774
KrVsKpEW	**0.0232**	0.0345	0.0301	0.0259	0.0393	0.0457	0.03	0.032	0.032	0.0292	0.0343	0.0391
BreastEW	**0.0368**	0.0386	0.0374	0.038	0.0396	0.0441	0.0381	0.0391	0.038	0.0384	0.039	0.0457
Lymphography	**0.1631**	0.1927	0.1689	0.1692	0.1996	0.2257	0.1825	0.1859	0.1826	0.1889	0.1762	0.2104
Vote	0.0026	0.007	**0.0022**	0.0031	0.0068	0.0181	0.0032	0.0076	0.0034	0.0049	0.0047	0.0206
Zoo	0.0033	0.0037	**0.0032**	0.0034	0.0039	0.0096	0.0037	0.0041	0.0038	0.0087	0.0038	0.0056
Exactly2	**0.2093**	0.2257	0.2121	0.2206	0.2331	0.2457	0.2244	0.2303	0.2274	0.2361	0.2308	0.2397
M-of-n	**0.0047**	0.0343	0.005	0.0075	0.0449	0.0711	0.0095	0.0111	0.0112	0.008	0.0091	0.062
WineEW	**0.0031**	0.0035	**0.0031**	0.0034	0.007	0.0183	0.0032	0.0035	0.0037	0.0035	0.0033	0.0084
BreastCancer	**0.0201**	0.0212	**0.0201**	**0.0201**	0.0233	0.0253	0.0203	0.0222	0.0219	0.0216	0.0211	0.0235
Tic-tac-toe	**0.1544**	**0.1544**	**0.1544**	**0.1544**	0.1602	0.1719	**0.1544**	**0.1544**	0.1552	0.1600	**0.1544**	0.1600
W Score T L	**9**	0	2	0	0	0	0	0	0	0	0	0
	**3**	1	3	2	0	0	1	1	0	0	1	0
	**2**	13	9	12	14	14	13	13	14	14	13	14

Superior values are in [bold].

**Table 9:** Results comparison of the mean accuracy (Mean_*Acc*_) based on the K-NN classifier for DESFO with others. MH methods.

Benchmarks	DESFO	DE	SFO	ABC	PSO	BA	GWO	WOA	GOA	HHO	BSA	HGSO
PenglungEW	**0.6533**	0.6067	0.6467	0.6267	0.6067	0.6067	0.6267	0.6200	0.6133	0.6200	0.6067	0.6000
IonosphereEW	**0.9324**	0.9141	0.9239	0.9197	0.907	0.8944	0.9155	0.9183	0.9183	0.9282	0.9155	0.8930
SonarEW	**0.9857**	0.9667	0.9833	0.9786	0.969	0.9429	0.9762	0.969	0.9738	0.9786	0.9762	0.9524
WaveformEW	**0.848**	0.8312	0.8416	0.8403	0.8268	0.8267	0.8386	0.8337	0.8359	0.8417	0.8359	0.8268
KrVsKpEW	**0.9822**	0.9714	0.9764	0.9802	0.9666	0.9595	0.9759	0.9736	0.9739	0.9762	0.9716	0.9675
BreastEW	**0.9649**	**0.9649**	**0.9649**	**0.9649**	0.964	0.9596	**0.9649**	0.964	**0.9649**	0.964	0.964	0.9588
Lymphography	**0.8400**	0.8100	0.8333	0.8333	0.8033	0.7767	0.82	0.8167	0.8200	0.8133	0.8267	0.7933
Vote	**1.0000**	0.9967	**1.0000**	**1.0000**	0.9967	0.985	**1.0000**	0.995	**1.0000**	0.9983	0.9983	0.9833
Zoo	**1.0000**	**1.0000**	**1.0000**	**1.0000**	**1.0000**	0.9952	**1.0000**	**1.0000**	**1.0000**	0.9952	**1.0000**	**1.0000**
Exactly2	**0.7935**	0.7765	0.7905	0.783	0.769	0.756	0.7785	0.773	0.7755	0.7665	0.7725	0.7640
M-of-n	**1.0000**	0.9715	**1.0000**	0.9975	0.961	0.9345	0.9955	0.994	0.994	0.997	0.996	0.9440
WineEW	**1.0000**	**1.0000**	**1.0000**	**1.0000**	0.9972	0.9861	**1.0000**	**1.0000**	**1.0000**	**1.0000**	**1.0000**	0.9972
BreastCancer	**0.9857**	**0.9857**	**0.9857**	**0.9857**	0.9829	0.9807	**0.9857**	0.9843	0.9843	0.9843	0.985	0.9836
Tic-tac-toe	**0.8542**	**0.8542**	**0.8542**	**0.8542**	0.8474	0.8344	**0.8542**	**0.8542**	0.8531	0.8469	**0.8542**	0.8474
W Score T L	**7**	0	0	0	0	0	0	0	0	0	0	0
	**7**	5	7	6	1	0	6	3	4	1	3	1
	**0**	9	7	8	13	14	8	11	10	13	11	13

Superior values are in [bold].

From Table 9, it is essential to note that DESFO-K-NN outperformed all other methods regarding accuracy mean values across seven benchmarks. In the remaining seven benchmarks, results were similar to those achieved by the different methods. DESFO-K-NN also showed significantly better performance in all 14 benchmarks, accounting for 100% of all benchmarks, which is a remarkable improvement compared to other methods. Additionally, In Table 10, a comparison of the mean number of selected features between the DESFO-K-NN method and other established meta-heuristic optimization algorithms is given. This comparison helps us understand the effectiveness of the DESFO-K-NN method in addressing the FS strategy.

**Table 10:** Results comparison of the mean number of features selected (Mean_Feat_) based on the K-NN classifier for DESFO with other MH methods.

Benchmarks	DESFO	DE	SFO	ABC	PSO	BA	GWO	WOA	GOA	HHO	BSA	HGSO
PenglungEW	**108.2**	149.2	149.9	150.6	153.7	159.5	148.2	151	152.4	131.8	147.6	167.2
IonosphereEW	**9.700**	12.70	10.40	12.50	12.90	14.20	12.20	12.6	13.50	10.9	12.00	17.70
SonarEW	23.60	28.10	24.40	27.40	30.20	24.60	26.40	26.1	27.30	**22.2**	24.60	32.40
WaveformEW	23.20	25.00	24.50	24.20	22.50	25.70	23.80	**21.3**	23.00	23.7	23.90	23.90
KrVsKpEW	**20.10**	22.20	24.20	22.40	22.40	20.20	22.10	21.2	22.10	20.3	22.30	25.10
BreastEW	**6.300**	11.70	8.000	9.900	12.10	12.50	10.00	10.4	9.800	8.50	10.30	14.70
Lymphography	8.500	8.300	**7.100**	7.600	8.900	8.300	7.800	7.90	8.000	7.30	8.200	10.50
Vote	4.100	5.900	**3.500**	4.900	5.600	5.200	5.100	4.20	5.500	5.20	4.900	6.500
Zoo	5.200	5.900	**5.100**	5.500	6.300	7.800	5.900	6.50	6.000	6.30	6.000	9.000
Exactly2	6.300	5.800	6.100	7.500	5.700	**5.400**	6.700	7.20	6.700	6.40	7.200	7.900
M-of-n	**6.100**	7.900	6.500	6.500	8.200	8.100	6.500	6.70	6.900	6.60	6.700	8.500
WineEW	**4.000**	4.500	**4.000**	4.400	5.500	5.900	4.200	4.60	4.800	4.50	4.300	7.400
BreastCancer	**6.000**	7.100	**6.000**	**6.000**	6.300	6.200	6.200	6.60	6.300	**6.00**	6.300	7.200
Tic-tac-toe	9.000	9.000	9.000	9.000	8.200	**7.100**	9.000	9.00	8.800	7.600	9.000	8.000
W Score T L	**5**	0	3	0	0	2	0	0	0	1	0	0
	**2**	0	2	1	0	0	0	1	0	1	0	0
	**7**	14	9	13	14	12	14	13	14	12	14	14

Superior values are in [bold].

Based on the results shown in Table 10, it can be inferred that the DESFO-K-NN algorithm has better exploration capabilities compared to other algorithms, as it has the lowest mean selected features number among all the algorithms tested (winning in 5 out of 7 cases and tying in 2 cases). This performance is superior to DE, PSO, GWO, GOA, BSA, and HGSO algorithms. It is worth mentioning that even though SFO selected fewer irrelevant features compared to DESFO-K-NN and other methods on only a few benchmarks (lymphography, vote, and Zoo), and achieved the same performance as DESFO-K-NN on two benchmarks (WineEw and BreastCancer), it did not outperform DESFO-K-NN in terms of mean accuracy. When selecting a minimal number of characteristics for classification, it is important to note that this approach can harm accuracy. The DESFO-K-NN algorithm has been proposed to efficiently identify the pertinent attributes and reduce the feature search area without compromising the classification accuracy. The algorithm achieves optimal results by discarding insignificant search areas and concentrating on the most viable ones.

### Analysis and visualization

**Figure 3:**
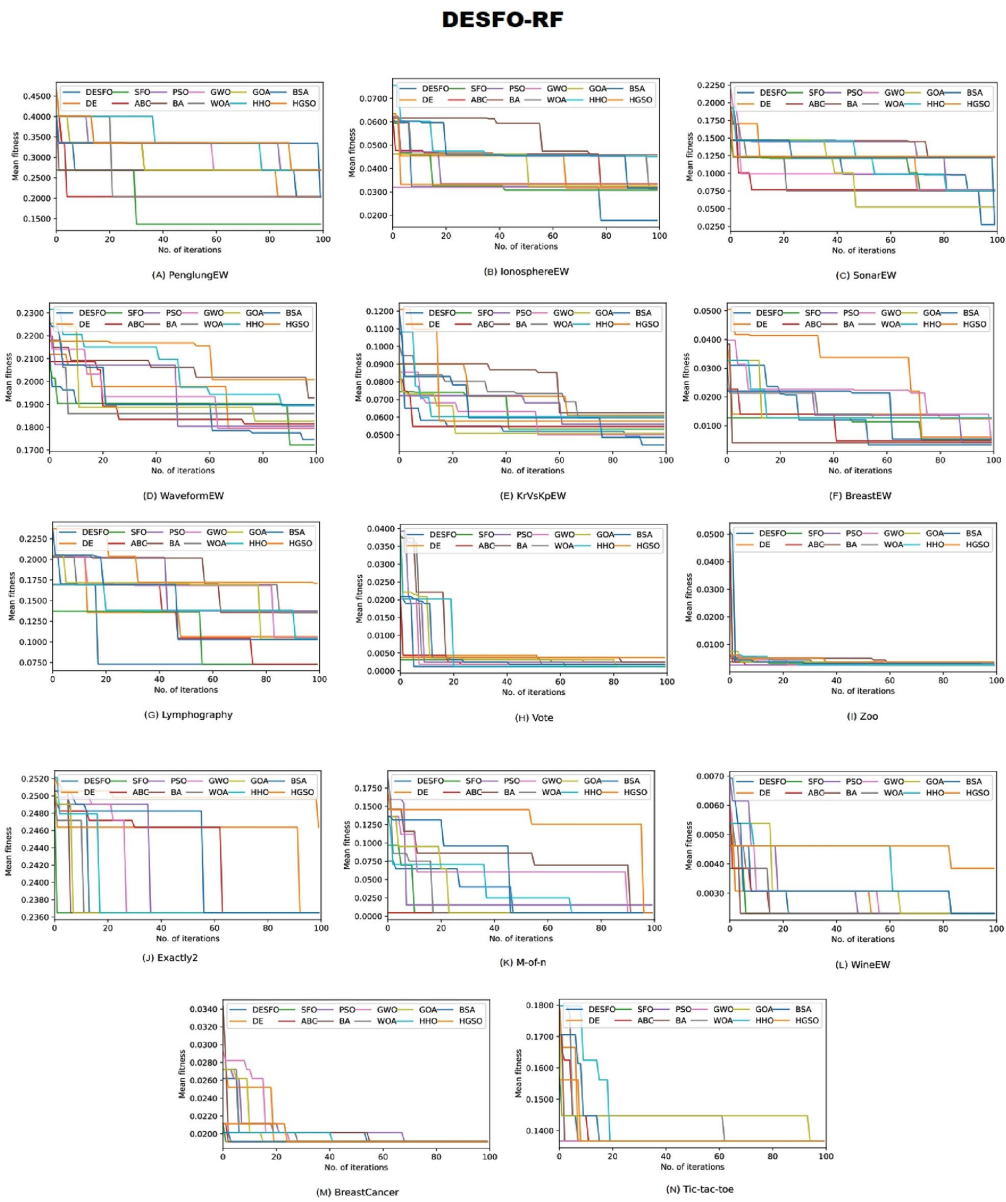
The convergence graphs comparing the suggested DESFO approach with other methods using the RF Classifier.

**Figure 4:**
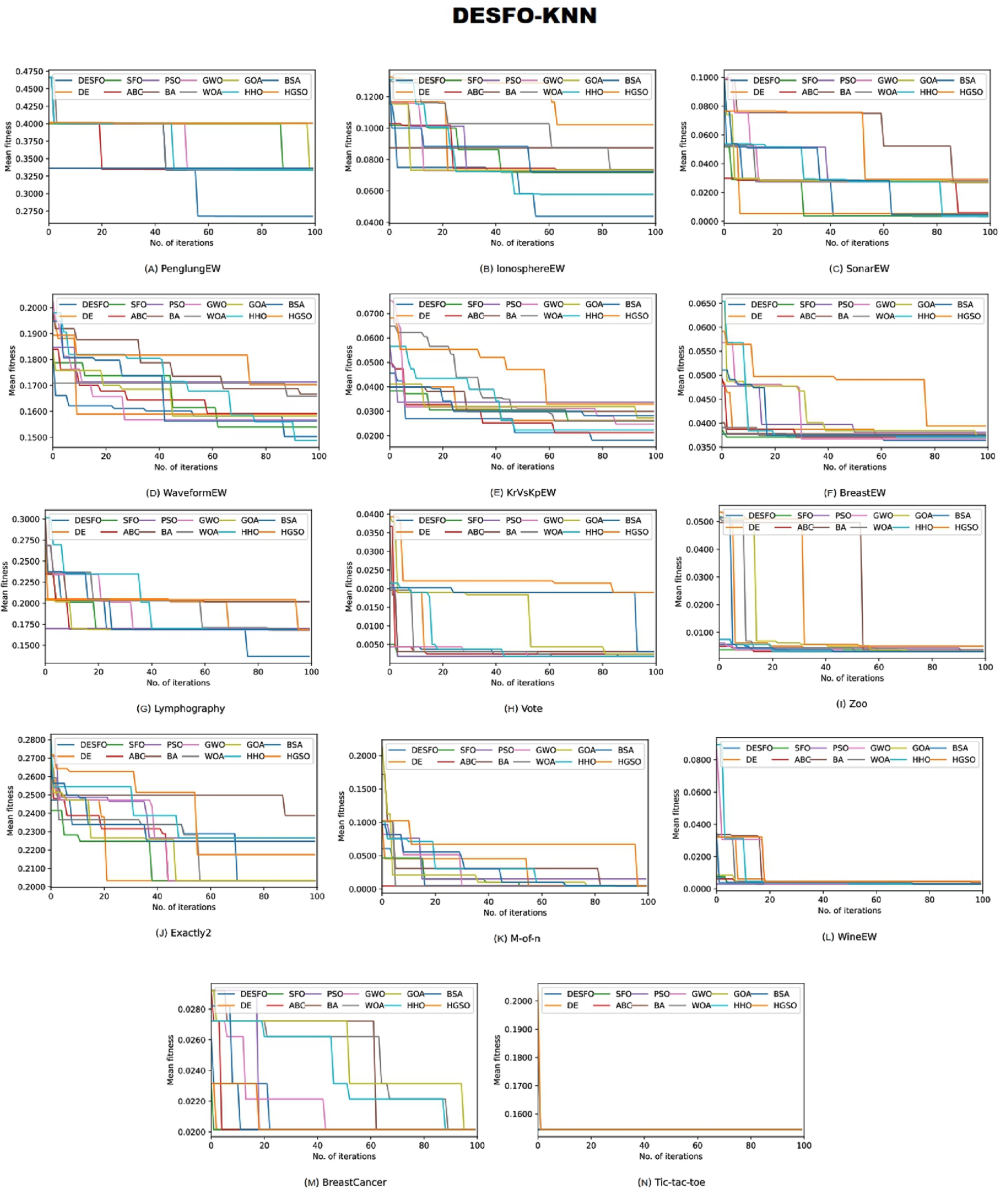
The convergence graphs comparing the suggested DESFO approach with other methods using the K-NN Classifier.

An analysis for DESFO–RF and DESFO-K-NN, used for handling the FS strategy, has been performed in this section using asymptotic analysis. To validate their convergence capabilities, the proposed technique was applied to 14 widely used benchmark datasets, and their performance has been compared against their peers under identical conditions, including the iteration number and population size. Figures 3 and 4 demonstrate the convergence ability of these methods in comparison to their counterparts.

Based on the results depicted in Fig. 3, the DESFO-RF approach showcases rapid yet effective convergence across eight benchmarks, including PenglungEW, IonosphereEW, SonarEW, WaveformEW, KrVsKpEW, BreastEW, Zoo, and Exactly2. On the other hand, Fig. 4 highlights that the DESFO-K-NN model outperforms the competition in five benchmarks, namely PenglungEW, IonosphereEW, SonarEW, WaveformEW, KrVsKpEW, BreastEW, Lymphography, Exactly2, and Lymphography. It’s worth noting that both the proposed algorithms (DESFO-RF and DESFO-K-NN) balance exploration and exploitation, ensuring the timely acquisition of the optimal solution.

**Figure 5:**
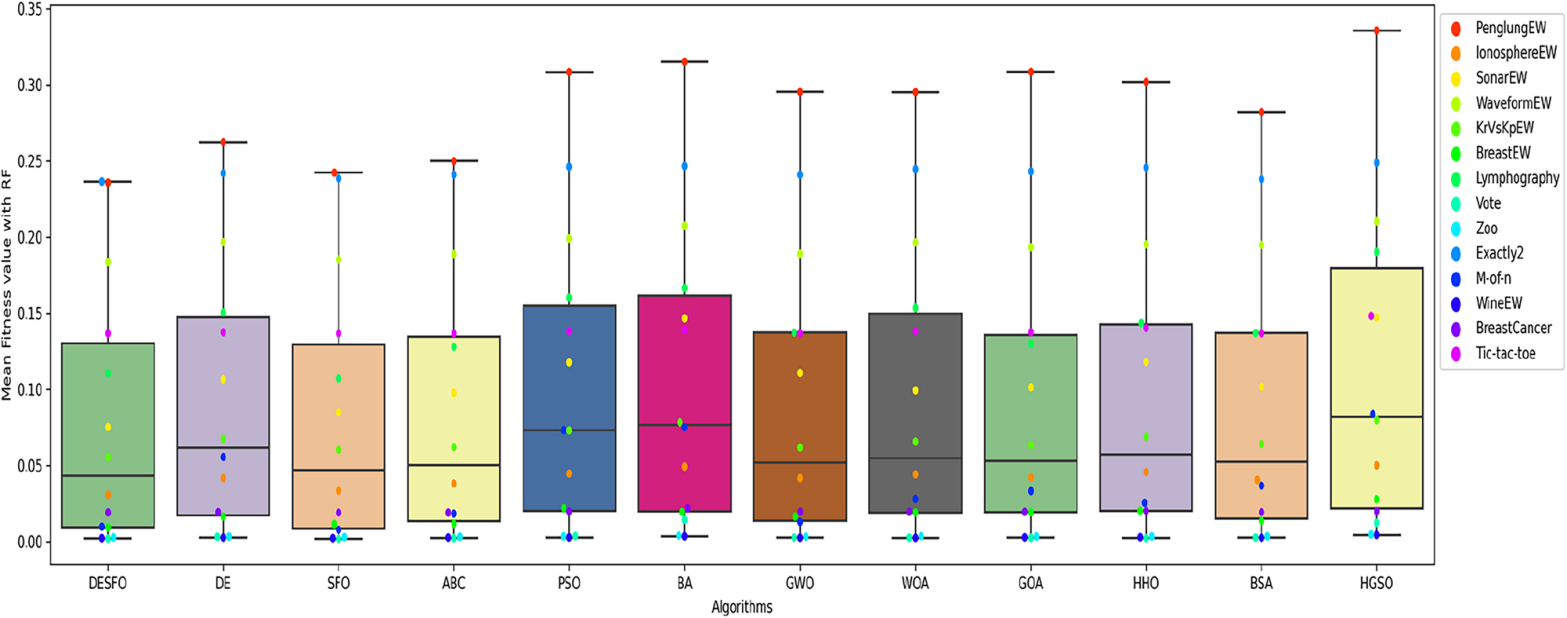
Box and swarm plot of DESFO-RF and Algorithms performance in terms of fitness value.

**Figure 6:**
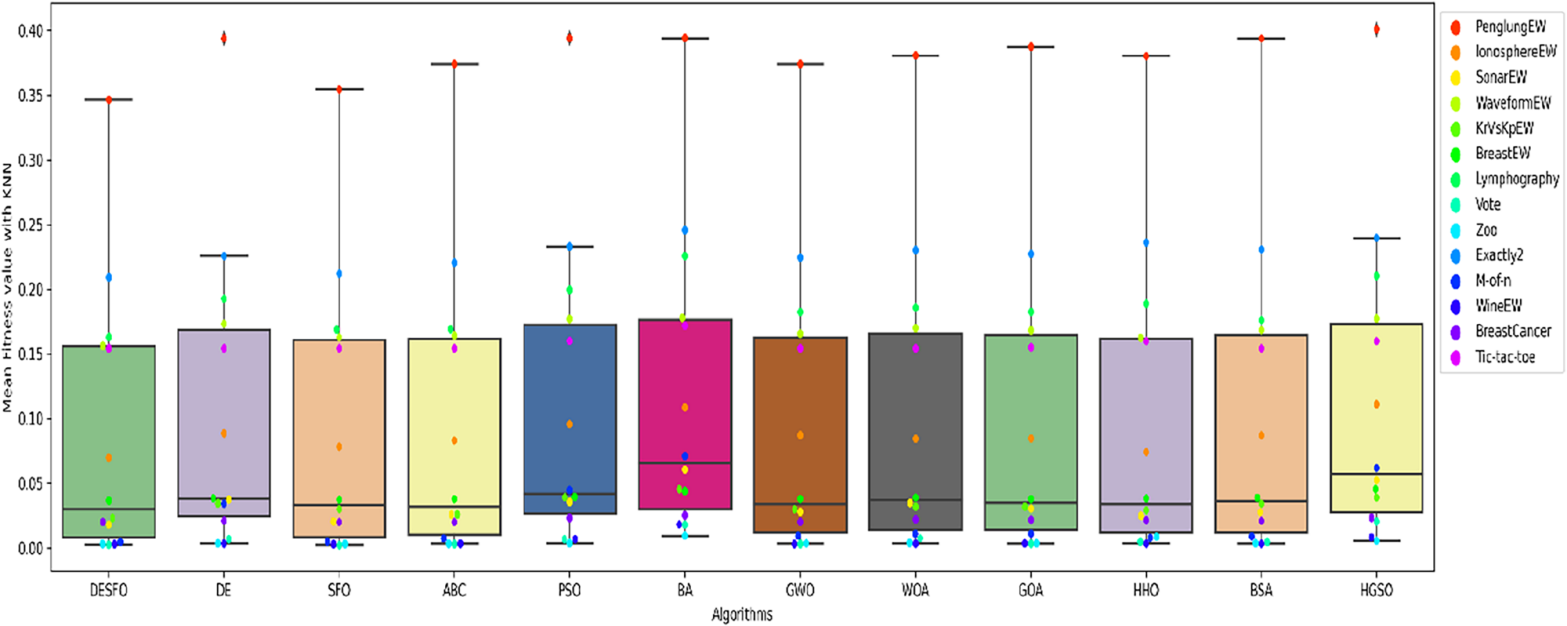
Box and swarm plot of DESFO-K-NN other Algorithms performance in terms of fitness value.

**Figure 7:**
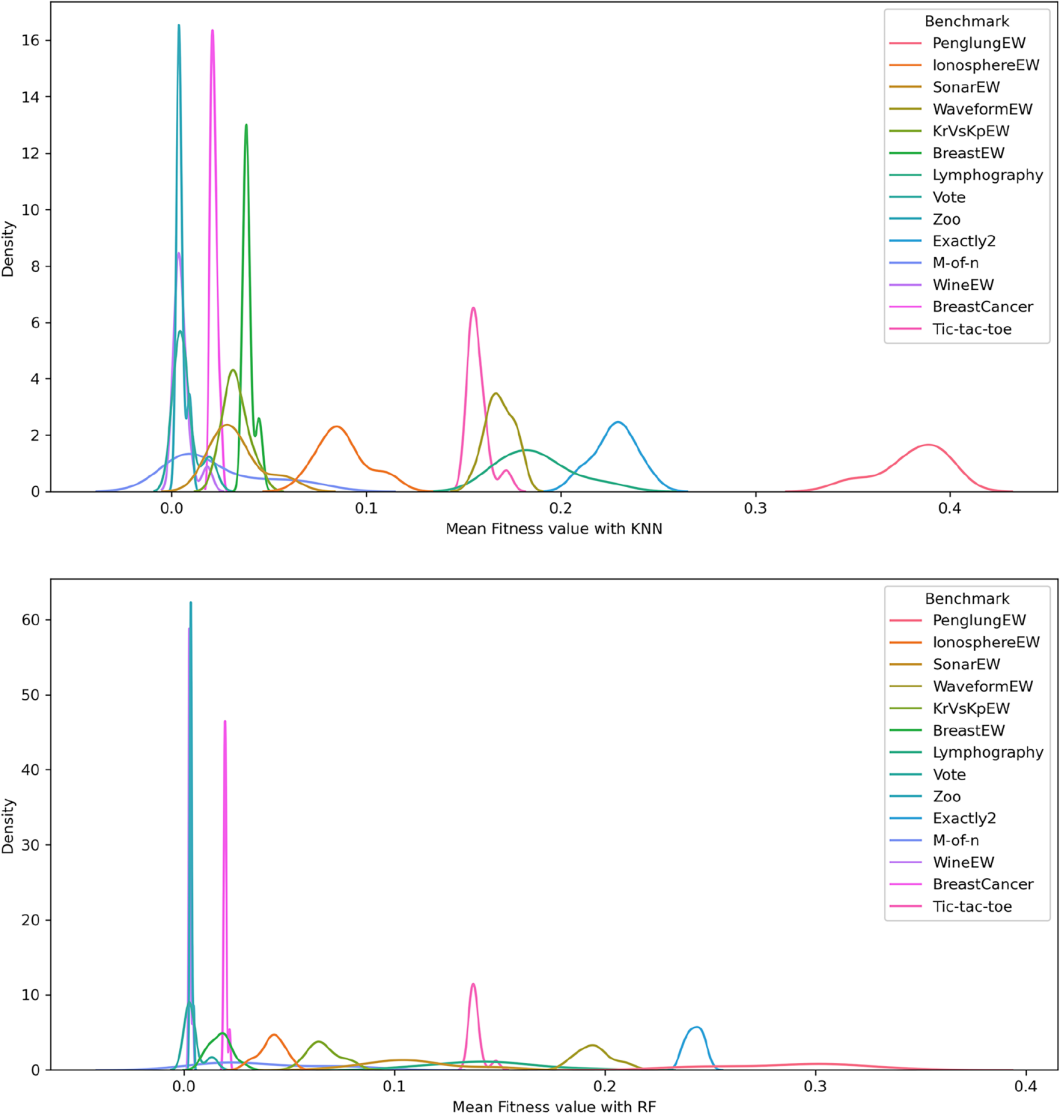
KDE plot diagram of DESFO and other Algorithms performance in terms of fitness value.

 Figures 5, 6, and 7 show the performance of DSEFO and other methods regarding Mean fitness Function values with RF and K-NN. The box plot with the swarm plot is demonstrated in Figs. 5 and 6, showing the superiority of DESFO over other algorithms. The plots reveal no outliers with Both DESFO-RF and DESFO-K-NN, unlike the DE, PSO, and HGSO Algorithms. The swarm plot demonstrates that most values are in the boxplot's interquartile range (IQR). Figure 7 shows the KDE plots, demonstrating the performance of DESFO and the other algorithms with the 14 UCI benchmarks.

**Figure 8:**
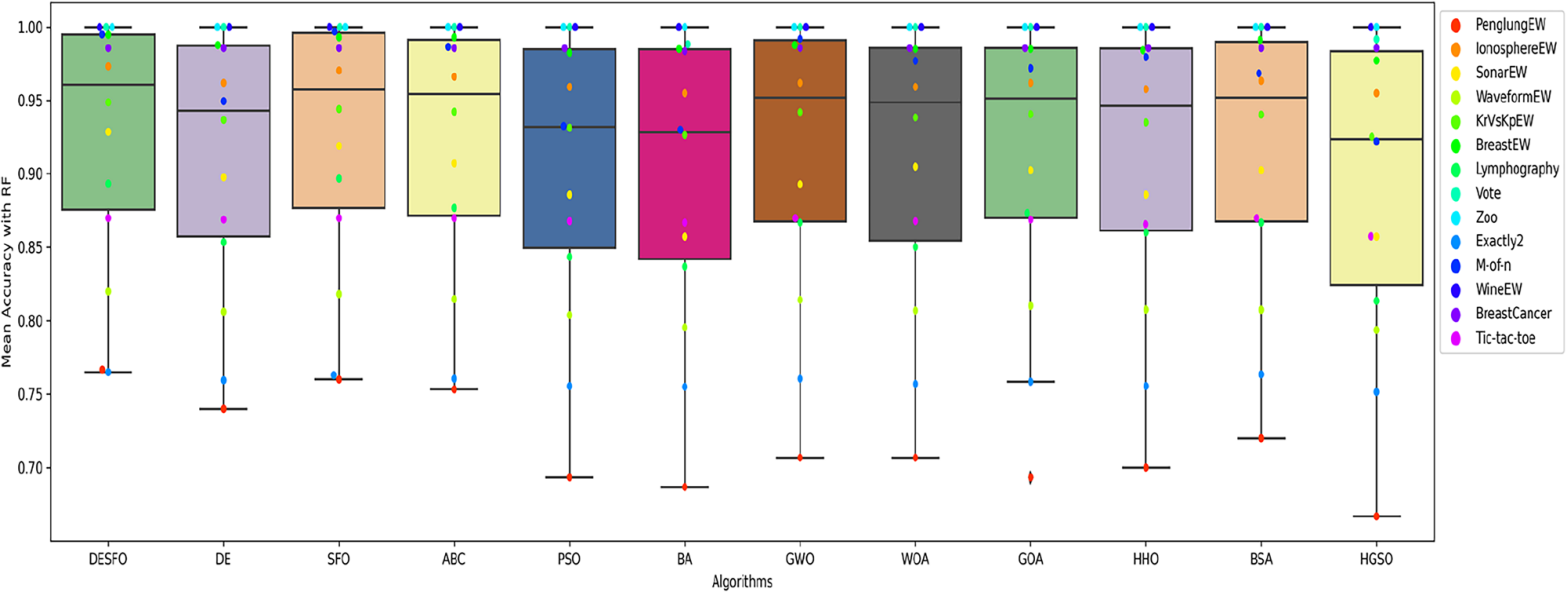
Box and swarm plot of DESFO-RF and Algorithms performance in term of Classification Accuracy.

**Figure 9:**
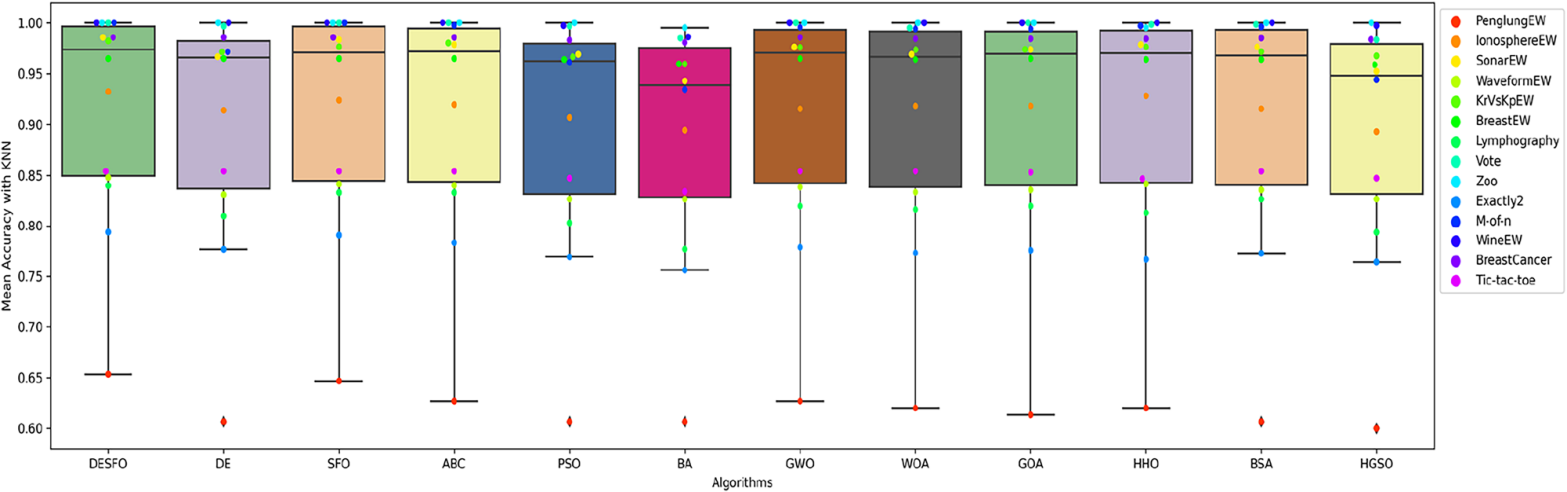
Box and swarm plot of DESFO-K-NN and Algorithms performance in term of classification accuracy.

**Figure 10:**
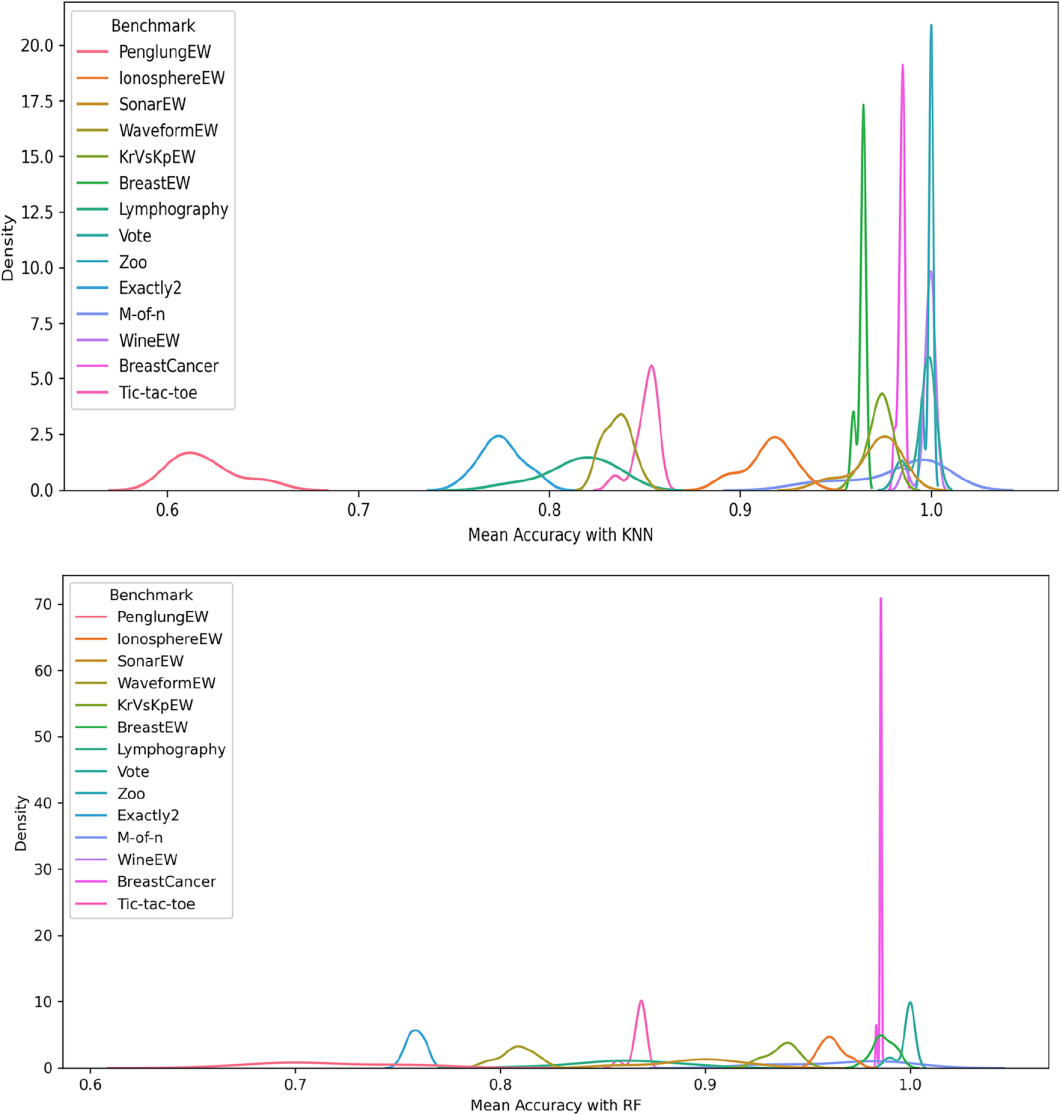
KDE plot diagram of DESFO and other Algorithms performance in term of classification accuracy.

Figures 8, 9, and 10 show the performance of DSEFO and other methods regarding Mean classification accuracy with RF and K-NN. Figures 8 and 9 illustrate the box plot with the swarm plot, highlighting the superior performance of DESFO over other algorithms. A noticeable observation from the plots is that no outliers exist in DESFO-RF and DESFO-K-NN, unlike other algorithms such as DE, PSO, BA, BSA, GOA, and HGSO Algorithms. The swarm plot indicates that for DESFO with RF and KNN, most of the values are located in the interquartile range (IQR) and the maximum value of the boxplot. Additionally, Fig. 10 shows KDE plots that depict the performance of DESFO and other algorithms with the 14 UCI benchmarks.

### Wilcoxon’s analysis

The statistical significance of the analysis can be observed in Tables 11 and 12, where the Wilcoxon test was conducted as a pair-wise assessment. This test helped to determine if there was a significant difference between the fitness results achieved by the proposed DESFO algorithm and its counterparts^74^.

**Table 11:** Wilcoxon’s test for DESO-RF vs Other algorithms.

DESFO-RF vs	$R^{{\left\{ + \right\}}}$	$R^{{\left\{ - \right\}}}$	Exact *P* value	Winner
DE	105	0	1.255E−04	DESFO
SFO	77.5	27.5	0.1270800	DESFO
ABC	90	1	4.88E−04	DESFO
PSO	105	0	1.22E−04	DESFO
BA	105	0	1.22E−04	DESFO
GWO	91	0	2.44E−04	DESFO
WOA	105	0	1.22E−04	DESFO
GOA	105	0	1.22E−04	DESFO
HHO	105	0	1.22E−04	DESFO
BSA	91	0	2.44E−04	DESFO
HGSO	105	0	1.22E−04	DESFO

**Table 12:** Wilcoxon’s test for DESO-K-NN versus Other algorithms.

DESFO-K-NN vs	$R^{{\left\{ + \right\}}}$	$R^{{\left\{ - \right\}}}$	Exact *P* value	Winner
DE	91	0	2.44E−04	DESFO
SFO	81.5	9.5	0.009277	DESFO
ABC	103.5	105	3.05E−04	DESFO
PSO	105	0	1.22E−04	DESFO
BA	105	0	1.22E−04	DESFO
GWO	91	0	2.44E−04	DESFO
WOA	91	0	2.44E−04	DESFO
GOA	105	0	1.22E−04	DESFO
HHO	105	0	1.22E−04	DESFO
BSA	91	0	2.44E−04	DESFO
HGSO	105	0	1.22E−04	DESFO

The Wilcoxon test is a statistical test often used in hypothesis testing situations. The test involves ranking the differences between the results of two paired algorithms on a set of problems. The calculation of ranks is based on the absolute values of the differences. Next, the positive and negative ranks are summed separately as R^+^ and R^−^. The smaller sum between the two is recorded. If the significance level of the recorded results is less than 5%, then the null hypothesis is rejected. On the other hand, if the significance level is greater than 5%, then the null hypothesis is not rejected.

After analyzing the data presented in Tables 11 and 12, it can be concluded that the DESFO-RF and DESFO-k-NN algorithms outperformed all other algorithms in all the tested scenarios. In Tables 11 and 12, the indicated *p* values are below 5%, implying that the proposed method’s results are statistically significant. This strong evidence against the null hypothesis suggests that the outcomes obtained are not due to chance.

### Discussion

According to the results of the empirical analysis, the DESFO algorithm stands out among recent algorithms in terms of its reliability in feature selection for classification tasks. This algorithm makes use of k-NN and RF classifiers. Among all the benchmarks, DESFO-K-NN produced the best results in terms of mean accuracy, followed by DESFO-RF. Additionally, the DESFO optimizer demonstrated a more pronounced exploration and exploitation behavior than its counterparts. On the other hand, The DESFO method exhibits a limitation in that it selects more features than its competitors across various datasets. Specifically, when compared with other methods, DESFO–RF selects a greater number of features in 9 out of 18 datasets (PenglungEW, IonosphereEW, WaveformEW, KrVsKpEW, Lymphography, Vote, Exacly2, BreastCancer, and Tic-tac-toe), while DESFO–K-NN does so in 7 datasets (SonarEW, WaveformEW, Lymphography, Vote, Zoo, Exactly2, and Tic-tac-toe).

## Conclusion and future works

The DESFO algorithm, a combination of the DE and SFO algorithms, has been proposed in this paper to handle FS strategies. The LS strategy has also been incorporated to improve the optimal results after each algorithm iteration. The algorithm has exhibited satisfactory performance and capability with significantly enhanced results. To evaluate the chosen feature subsets, RF and K-NN classifiers were used to calculate the classification accuracy. The DESFO algorithm was tested on several benchmarks using multi-scale attributes and records in this work to assess its effectiveness. The results were compared with binary versions of 11 different meta-heuristic methods. The performance has been evaluated based on various metrics, such as mean fitness rate, mean accuracy rate, and mean number of features selected. The findings indicated that the two algorithms proposed in the study (DESFO–RF and DESFO–K-NN) outperformed their counterparts in managing FS strategies. DESFO-RF was the most effective method among all benchmarks regarding mean accuracy results, followed by IBAO-k-NN.

Additionally, the DESFO optimizer demonstrated greater exploration and exploitation abilities than its counterparts. According to Wilcoxon's test (with a significance level of α = 0.05), it was evident that the DESFO algorithm with RF and k-NN classifiers outperformed the other methods. This algorithm achieved exceptional classification accuracy up to 100% in some benchmarks and also resulted in a reduced feature size.

The DESFO technique has one limitation: it tends to choose more features than its rivals across different datasets. Specifically, in comparison with other methods, DESFO–RF selects more features in 9 out of 18 datasets, and DESFO–K-NN does so in 7 datasets. Therefore, to improve the proposed algorithm, it would be beneficial to implement a new selection strategy to reduce the number of features selected, particularly for high-dimensional datasets with small instances. This opens up avenues for further research in the future.

Integrating the DESFO algorithm with various other optimization techniques merits exploration for future works. Additionally, the application of different classifiers, such as Artificial Neural Networks (ANNs), Decision Trees (DT), support vector machines (SVM), and others, could further examine DESFO’s capability in feature selection for classification. The adaptation of other transfer functions, such as S-shape functions, could also be explored. Given its feature selection (FS) efficacy, DESFO presents significant potential across various domains, such as healthcare, the Internet of Things (IoT), and intrusion detection systems. Furthermore, employing DESFO in the context of CEC benchmark functions could also be explored.

## Data Availability

The datasets used in our research are available and stored in a public access repository designed for machine learning purposes and data classification which is the UC Irvine Machine Learning Repository https://archive.ics.uci.edu/datasets, it is important to be declared that we used 14 variant datasets, include: IonosphereEW from the link: https://archive.ics.uci.edu/dataset/52/ionosphere). Waveform from the link: (https://archive.ics.uci.edu/dataset/108/waveform+database+generator+version+2). lymphography from the link: https://archive.ics.uci.edu/dataset/63/lymphography. Zoo from https://archive.ics.uci.edu/dataset/111/zoo. Breastcancer from the link: https://archive.ics.uci.edu/dataset/15/breast+cancer+wisconsin+original. BreastEW from the link https://archive.ics.uci.edu/dataset/17/breast+cancer+wisconsin+diagnostic. Tic Tac Toe from the link: https://archive.ics.uci.edu/dataset/101/tic+tac+toe+endgame. SonarEW from the link: https://archive.ics.uci.edu/dataset/151/connectionist+bench+sonar+mines+vs+rocks. Wine from the link: https://archive.ics.uci.edu/dataset/109/wine. KrVsKpEW from the link: https://archive.ics.uci.edu/dataset/22/chess+king+rook+vs+king+pawn. The rest of them can be accessed directly from GitHub link: https://github.com/trin07/MA-HS/commit/3fea0b1c4470170f3408cedab76a3e82493b3b5f.

## References

[1] Abd El-Mageed AA, Abohany AA, Elashry A (2023). Effective feature selection strategy for supervised classification based on an improved binary Aquila optimization algorithm. Comput. Ind. Eng..

[2] Liu H, Motoda H (1998). Feature Extraction, Construction and Selection: A Data Mining Perspective.

[3] Faris H (2018). An efficient binary Salp Swarm Algorithm with crossover scheme for feature selection problems. Knowl.-Based Syst..

[4] Barddal JP, Enembreck F, Gomes HM, Bifet A, Pfahringer B (2019). Merit-guided dynamic feature selection filter for data streams. Expert Syst. Appl..

[5] González J, Ortega J, Damas M, Martín-Smith P, Gan JQ (2019). A new multi-objective wrapper method for feature selection – Accuracy and stability analysis for BCI. Neurocomputing.

[6] Zhang R, Nie F, Li X, Wei X (2019). Feature selection with multi-view data: A survey. Inf. Fus..

[7] Zhigljavsky, A. A. *Theory of Global Random Search (Mathematics and its Applications)*. (1991) [Online]. Available: http://www.amazon.ca/exec/obidos/redirect?tag=citeulike09-20&path=ASIN/0792311221%5Cnhttp://www.amazon.de/exec/obidos/redirect?tag=citeulike01-21&path=ASIN/0792311221%5Cnhttp://www.amazon.fr/exec/obidos/redirect?tag=citeulike06-21&path=ASIN/07

[8] Amaldi E, Kann V (1998). On the approximability of minimizing nonzero variables or unsatisfied relations in linear systems. Theor. Comput. Sci..

[9] Khurma RA, Aljarah I, Sharieh A (2021). A simultaneous moth flame optimizer feature selection approach based on levy flight and selection operators for medical diagnosis. Arab. J. Sci. Eng..

[10] Rodrigues D, Yang XS, De Souza AN, Papa JP (2015). Binary flower pollination algorithm and its application to feature selection. Stud. Comput. Intell..

[11] Karaboga D, Basturk B (2007). A powerful and efficient algorithm for numerical function optimization: Artificial bee colony (ABC) algorithm. J. Glob. Optim..

[12] Eberhart, R. & Sixth, J. K. A new optimizer using particle swarm theory. In: *Proceedings IEEE Symposium on Micromechatronics and Human Science Nagoys, Japan* 39–43 (1997) [Online]. Available: https://ieeexplore.ieee.org/abstract/document/494215.?casa_token=VRHbIOq0xY0AAAAA:tigoKrFPGIOWOZPL3HUCxeJDuwpHdMr7AdrNcyfXSzfY9zdeQ3AAVzx9vd-b63ZQ8Q1ZwFq8E5okfcE

[13] Li XL, Shao ZJ, Qian JX (2002). Optimizing method based on autonomous animats: Fish-swarm Algorithm. Xitong Gongcheng Lilun yu Shijian/System Eng Theory Pract..

[14] Passino, K. M., Biomimicry of bacterial foraging. *Small* 52–67 (2002).

[15] Dorigo M, Maniezzo V, Colorni A (1996). Ant system: Optimization by a colony of cooperating agents. IEEE Trans Syst. Man Cybern. Part B Cybern..

[16] Duan H, Qiao P (2014). Pigeon-inspired optimization: A new swarm intelligence optimizer for air robot path planning. Int. J. Intell. Comput. Cybern..

[17] Yang XS, Gandomi AH (2012). Bat algorithm: A novel approach for global engineering optimization. Eng. Comput. (Swansea, Wales).

[18] Mirjalili S, Mirjalili SM, Lewis A (2014). Grey wolf optimizer. Adv. Eng. Softw..

[19] Črepinšek M, Liu S-H, Mernik M (2013). Exploration and exploitation in evolutionary algorithms: A survey. ACM Comput. Surv..

[20] Morales-Castañeda B, Zaldivar D, Cuevas E, Fausto F, Rodríguez A (2020). A better balance in metaheuristic algorithms: Does it exist?. Swarm Evol. Comput..

[21] Li J, Gao L, Li X (2024). Multi-operator opposition-based learning with the neighborhood structure for numerical optimization problems and its applications. Swarm Evol. Comput..

[22] Shadravan S, Naji HR, Bardsiri VK (2019). The Sailfish Optimizer: A novel nature-inspired metaheuristic algorithm for solving constrained engineering optimization problems. Eng. Appl. Artif. Intell..

[23] Pierezan J, dos Santos Coelho L, CoccoMariani V, de Vasconcelos Segundo EH, Prayogo D (2021). Chaotic coyote algorithm applied to truss optimization problems. Comput. Struct..

[24] Almufti S (2021). The novel social spider optimization algorithm: Overview, modifications, and applications. Icontech Int. J..

[25] Klein, C. E., Mariani, V. C. & Coelho, L. D. S. Cheetah based optimization algorithm: A novel swarm intelligence paradigm. in *ESANN 2018 proceedings, European Symposium on Artificial Neural Networks, Computational Intelligence and Machine Learning* 685–690 (2018).

[26] Tongur V, Ertunc E, Uyan M (2020). Use of the Migrating Birds Optimization (MBO) Algorithm in solving land distribution problem. Land Use Policy.

[27] de Vasconcelos Segundo EH, Mariani VC, Coelho LS (2019). Metaheuristic inspired on owls behavior applied to heat exchangers design. Therm. Sci. Eng. Prog..

[28] Das S, Biswas A, Dasgupta S, Abraham A (2009). Bacterial foraging optimization algorithm: Theoretical foundations, analysis, and applications. Stud. Comput. Intell..

[29] Mirjalili S, Gandomi AH, Mirjalili SZ, Saremi S, Faris H, Mirjalili SM (2017). Salp Swarm algorithm: A bio-inspired optimizer for engineering design problems. Adv. Eng. Softw..

[30] Storn R, Price K (1997). Differential evolution–a simple and efficient heuristic for global optimization over continuous spaces. J. Glob. Optim..

[31] Holland JH (1992). Genetic algorithms. Sci. Am..

[32] Tang D, Dong S, Jiang Y, Li H, Huang Y (2015). ITGO: Invasive tumor growth optimization algorithm. Appl. Soft Comput. J..

[33] Simon D (2008). Biogeography-based optimization. IEEE Trans. Evol. Comput..

[34] Erol OK, Eksin I (2006). A new optimization method: Big Bang-Big crunch. Adv. Eng. Softw..

[35] Mirjalili S, Mirjalili SM, Hatamlou A (2016). Multi-verse optimizer: A nature-inspired algorithm for global optimization. Neural Comput. Appl..

[36] Rashedi E, Nezamabadi-pour H, Saryazdi S (2009). GSA: A gravitational search algorithm. Inf. Sci. (NY).

[37] Mahmoudi, S., Rajabioun, R. & Lotfi, S. Binary cuckoo optimization algorithm. *Nature*, 1–7 (2013).

[38] Emary, E., Zawbaa, H. M., Ghany, K. K. A., Hassanien, A. E. & Parv, B. Firefly optimization algorithm for feature selection. in *Proceedings of the 7th Balkan Conference on Informatics Conference* 1–7 (2015).

[39] Nakamura, R. Y. M., Pereira, L. A. M., Costa, K. A., Rodrigues, D., Papa, J. P., & Yang, X. S. BBA: A binary bat algorithm for feature selection. In: *Brazilian Symposium Computer Graphics and Image Processing* 291–297 10.1109/SIBGRAPI.2012.47. (2012).

[40] Zawbaa, H. M., Emary, E. & Parv, B. Feature selection based on antlion optimization algorithm. in *2015 Third World Conference on complex systems (WCCS)* 1–7 (IEEE, 2015).

[41] Emary E, Zawbaa HM, Hassanien AE (2016). Binary grey wolf optimization approaches for feature selection. Neurocomputing.

[42] Hussien G, Hassanien AE, Houssein EH, Bhattacharyya S, Amin M (2019). S-shaped Binary Whale Optimization Algorithm for Feature Selection.

[43] Hussien, A. G., Houssein, E. H. & Hassanien, A. E. A binary whale optimization algorithm with hyperbolic tangent fitness function for feature selection. in *2017 IEEE 8th International Conference on Intelligent Computing and Information Systems (ICICIS) 2017*, vol. 2018, pp. 166–172 10.1109/INTELCIS.2017.8260031 (2017).

[44] Gad AG, Sallam KM, Chakrabortty RK, Ryan MJ, Abohany AA (2022). An improved binary sparrow search algorithm for feature selection in data classification. Neural Comput. Appl..

[45] Ghosh KK, Ahmed S, Singh PK, Geem ZW, Sarkar R (2020). Improved binary sailfish optimizer based on adaptive β-Hill climbing for feature selection. IEEE Access.

[46] Hancer E, Xue B, Zhang M (2018). Differential evolution for filter feature selection based on information theory and feature ranking. Knowl.-Based Syst..

[47] Bacanin N (2023). Addressing feature selection and extreme learning machine tuning by diversity-oriented social network search: An application for phishing websites detection. Complex Intell. Syst..

[48] Alrefai N, Ibrahim O (2022). Optimized feature selection method using particle swarm intelligence with ensemble learning for cancer classification based on microarray datasets. Neural Comput. Appl..

[49] Gomez Y, Bello R, Puris A, Garcia MM, Nowe A (2008). Two step swarm intelligence to solve the feature selection problem. J. Univ. Comput. Sci..

[50] Bezdan T, Zivkovic M, Bacanin N, Chhabra A, Suresh M (2022). Feature selection by hybrid brain storm optimization algorithm for covid-19 classification. J. Comput. Biol..

[51] Gao J, Wang Z, Lei Z, Wang R-L, Zhengwei W, Gao S (2024). Feature selection with clustering probabilistic particle swarm optimization. Int. J. Mach. Learn. Cybern..

[52] Latha RS, Saravana Balaji B, Bacanin N, Strumberger I, Zivkovic M, Kabiljo M (2022). Feature selection using grey wolf optimization with random differential grouping. Comput. Syst. Sci. Eng..

[53] Ilonen J, Kamarainen J-K, Lampinen J (2003). Differential evolution training algorithm for feed-forward neural networks. Neural Process. Lett..

[54] Storn, R. On the usage of differential evolution for function optimization. in *Proceedings of North American fuzzy Information Processing* 519–523 (IEEE, 1996).

[55] Rogalsky T, Kocabiyik S, Derksen RW (2000). Differential evolution in aerodynamic optimization. Can. Aeronaut. Sp. J..

[56] Joshi R, Sanderson AC (1999). Minimal representation multisensor fusion using differential evolution. IEEE Trans. Syst. Man Cybern. A Syst. Humans.

[57] Frank, A. UCI machine learning repository. http//archive.ics.uci.edu/ml (2010).

[58] Wolpert DH, Macready WG (1997). No free lunch theorems for optimization. IEEE Trans. Evol. Comput..

[59] Sallam, K. M., Elsayed, S. M., Sarker, R. A. & Essam, D. L. Multi-method based orthogonal experimental design algorithm for solving CEC2017 competition problems. in *2017 IEEE Congress on Evolutionary Computation (CEC)* 1350–1357 (IEEE, 2017).

[60] Venkatesh B, Anuradha J (2019). A review of feature selection and its methods. Cybern. Inf. Technol..

[61] Zhang, W.-J., Xie, X.-F. & Bi, D.-C. Handling boundary constraints for numerical optimization by particle swarm flying in periodic search space. In *Proceedings of the 2004 Congress on Evolutionary Computation (IEEE Cat. No. 04TH8753)* 2307–2311 (IEEE, 2004).

[62] Alpaydin E (2020). Introduction to Machine Learning.

[63] Criminisi A, Shotton J, Konukoglu E (2012). Decision forests: A unified framework for classification, regression, density estimation, manifold learning, and semi-supervised learning. Found. Trends® Comput. Graph Vis..

[64] Tharwat A, Hassanien AE, Elnaghi BE (2017). A BA-based algorithm for parameter optimization of support vector machine. Pattern Recogn. Lett..

[65] Schölkopf B, Smola AJ (2002). Learning with Kernels: Support Vector Machines, Regularization, Optimization, and Beyond.

[66] Heidari AA, Mirjalili S, Faris H, Aljarah I, Mafarja M, Chen H (2019). Harris hawks optimization: Algorithm and applications. Futur. Gener. Comput. Syst..

[67] Kennedy, J. & Eberhart, R. Particle swarm optimization. in *Proceedings of ICNN’95-International Conference on Neural Networks* 1942–1948 (IEEE, 1995).

[68] Mirjalili S, Lewis A (2016). The whale optimization algorithm. Adv. Eng. Softw..

[69] Saremi S, Mirjalili S, Lewis A (2017). Grasshopper optimisation algorithm: Theory and application. Adv. Eng. Softw..

[70] Meng X-B, Gao XZ, Lu L, Liu Y, Zhang H (2016). A new bio-inspired optimisation algorithm: Bird Swarm Algorithm. J. Exp. Theor. Artif. Intell..

[71] Hashim FA, Houssein EH, Mabrouk MS, Al-Atabany W, Mirjalili S (2019). Henry gas solubility optimization: A novel physics-based algorithm. Futur. Gener. Comput. Syst..

[72] Abd El-Mageed AA, Gad AG, Sallam KM, Munasinghe K, Abohany AA (2022). Improved binary adaptive wind driven optimization algorithm-based dimensionality reduction for supervised classification. Comput. Ind. Eng..

[73] Zaki MJ, Meira W (2014). Data Mining and Analysis: Fundamental Concepts and Algorithms.

[74] Derrac J, García S, Molina D, Herrera F (2011). A practical tutorial on using nonparametric statistical tests as a methodology for comparing evolutionary and swarm intelligence algorithms. Swarm Evol. Comput..

